# Design and synthesis of *C*_3_-symmetric molecules bearing propellane moieties via cyclotrimerization and a ring-closing metathesis sequence

**DOI:** 10.3762/bjoc.14.230

**Published:** 2018-10-01

**Authors:** Sambasivarao Kotha, Saidulu Todeti, Vikas R Aswar

**Affiliations:** 1 Department of Chemistry, Indian Institute of Technology Bombay, Powai, Mumbai-400076, India

**Keywords:** cyclotrimerization, Diels–Alder, propellane, ring-closing metathesis

## Abstract

We have developed an efficient synthetic strategy to assemble *C*_3_-symmetric molecules containing propellane moieties as end groups and a benzene ring as a central core. The synthesis of these *C*_3_-symmetric molecules involves simple starting materials. Our approach to *C*_3_-symmetric compounds relies on a Diels–Alder reaction, cyclotrimerization and ring-closing metathesis as key steps.

## Introduction

In 1966 Ginsburg coined the word “propellane” [1–2] and Wiberg reviewed various aspects of medium and small ring propellanes [3–4]. Propellanes consist of tricyclic compounds where three rings are conjoined by a common C–C bond [1,5–6]. Heterocyclic systems contain a heteroatom (e.g., oxygen, nitrogen, and sulfur, etc.) along with carbon atoms. The name of a heterocyclic propellane may be organized by prefixing aza, oxa, etc.

**Figure 1:**
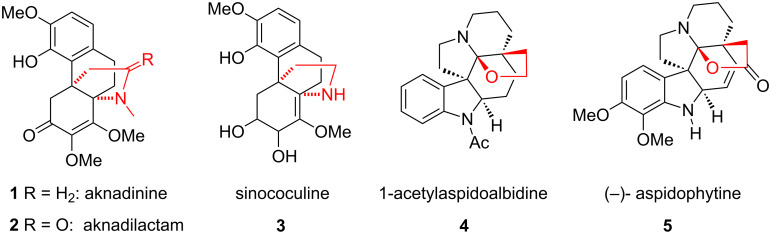
Various alkaloids containing propellane frame work.

Among various propellanes, nitrogen-containing compounds occupy a special place because they are present as core structural units in bioactive natural products and pharmaceuticals. Some of these propellanes exhibit intresting properties like antibiotic, antifungal, anticancer, platelet-activating factor antagonistic and antibacterial activities. The propellane skeleton is present in many alkaloids such as aknadinine (**1**), aknadilactam (**2**), and the known morphinane alkaloid sinococuline (**3**), which was identified as a bioactive component from *S. japonica* [7]. In 1963 Brown et al. isolated 1-acetyl-aspidoalbidine (**4**) from *Vallesia dichtoma* [8] and subsequently, Djerassi proposed its structure [9]. Another alkaloid (−)-aspidophytine (**5**) differs from 1-acetylaspidoalbidine (**4**) only in the degree of unsaturation and the substitution pattern on the aromatic ring (Figure 1).

The design of propellanes demands unique synthetic methods and these include: manganese or palladium-catalyzed transformations [10], the Diels–Alder (DA) reaction [11–12], and rearrangement of spiro-ketones, nucleophilic substitutions of alkenes, and photochemical addition reactions. Multicomponent reactions (MCRs) are also used for the synthesis of hetero-propellanes [13–14]. Recently, heterocyclic propellanes have been reviewed [15–16]. Our group also developed simple synthetic approaches to propellanes via ring-closing metathesis (RCM) as a key step [17–18].

The development of new synthetic strategies to *C*_3_-symmetric molecules bearing propellane moieties from commercially available starting materials is worthy of systematic investigation. To this end, our efforts are directed to design star-shaped molecules that involve a wide range of structural variations. To the best of our knowledge there are no synthetic reports available for *C*_3_-symmetric molecules bearing propellane moieties. As part of our major program aimed at designing star-shaped *C*_3_-symmetric molecules [19–30], here, we conceived new strategies to N-containing star-shaped molecules. Such star-shaped molecules are generally used in organic light-emitting diodes (OLEDs) [31–33], organic photovoltaics (OPVs) [34], organic field-effect transistors (OFETs) [35–36], and other optoelectronic devices. Our approach to *C*_3_-symmetric molecules containing propellane moieties involve DA reaction [37], cyclotrimerization [19] and RCM [38–41] as key steps.

## Results and Discussion

**Scheme 1:**
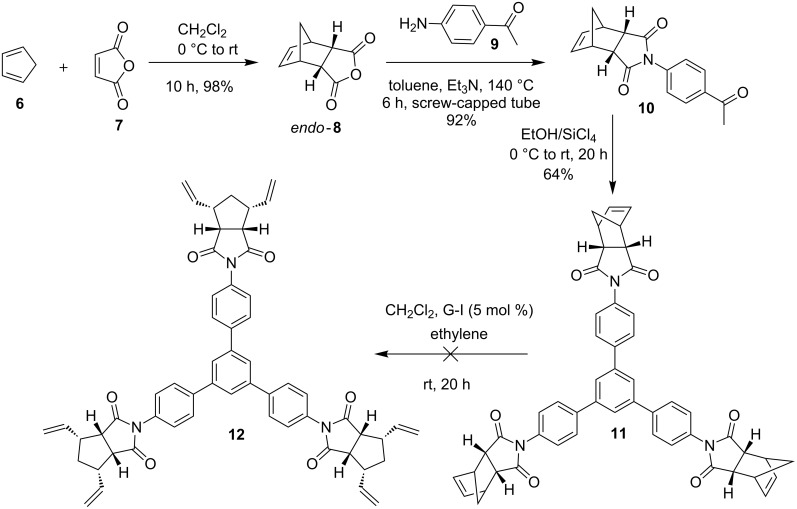
Synthesis of the star-shaped norbornene derivative **11** via trimerization.

The synthesis of propellane-bearing *C*_3_-symmetric derivatives starts with commercially available dicyclopentadiene and maleic anhydride (**7**). Here, we used a DA reaction of freshly cracked cyclopentadiene (**6**) and maleic anhydride (**7**) to obtain the *endo*-DA adduct **8** [42] in 98% yield. Next, this cycloadduct **8** was treated with commercially available 4-aminoacetophenone (**9**) in the presence of triethylamine (Et_3_N) in toluene at 140 °C to obtain the acetophenone derivative **10** in excellent yield (92%) [43]. Later, the acetophenone derivative **10** was subjected to trimerization reaction under ethanol/silicon tetrachloride (EtOH/SiCl_4_) conditions to deliver the trimerized product **11** (64%). Having the trimerized product **11**, we attempted to open the norbornene system due to the fact that not all norbornene rings open up during RCM to generate propellane derivative. After allylation, RCM is not a clean reaction and it gave a mixture of the *C*_3_-symmetrical compounds. Therefore it is desirable to open the norbornene double bond before the trimerization sequence. To this end, the trimerized product **11** was treated with Grubbs first generation (G-I) catalyst in CH_2_Cl_2_ under ethylene atmosphere but, we were unable to get the ring-opened product **12** (Scheme 1).

**Figure 2:**
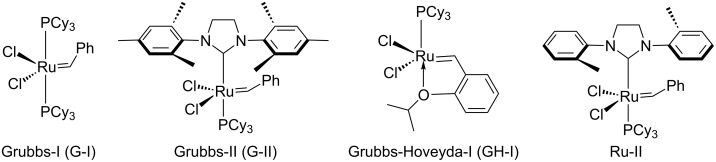
Selected list of ruthenium-based catalysts used for ROM.

Later, we considered an alternate route to synthesize compound **12**. In this regard, we employed different ruthenium-based catalysts (Figure 2) and reaction conditions to obtain the ring-opening metathesis (ROM) product **13** from norbornene derivate **10**. Under these conditions the starting material was not consumed completely. After some experimentation, we found that G-I catalyst (5 mol %) in CH_2_Cl_2_ is suitable to generate the ROM product **13** in 56% yield (Table 1).

**Table 1:** Different conditions attempted to obtain the ROM product **13**.

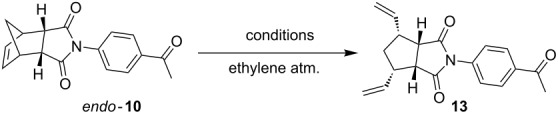						

entry	catalyst	mol %	solvent	temp	time (h)	yield (%)

1	G-I	5 or 10	CH_2_Cl_2_	rt	48	56
2	G-I	5 or 10	CH_2_Cl_2_	reflux	32	48
4	G-II	5 or 10	toluene	rt	46	24
5	G-II	5	toluene	reflux	43	20
6	Ru-II	5 or 10	CH_2_Cl_2_	rt	48	52
7	GH-I	5 or 10	CH_2_Cl_2_	rt	40	53
8	GH-I	5 or 10	CH_2_Cl_2_	reflux	40	50

**Scheme 2:**
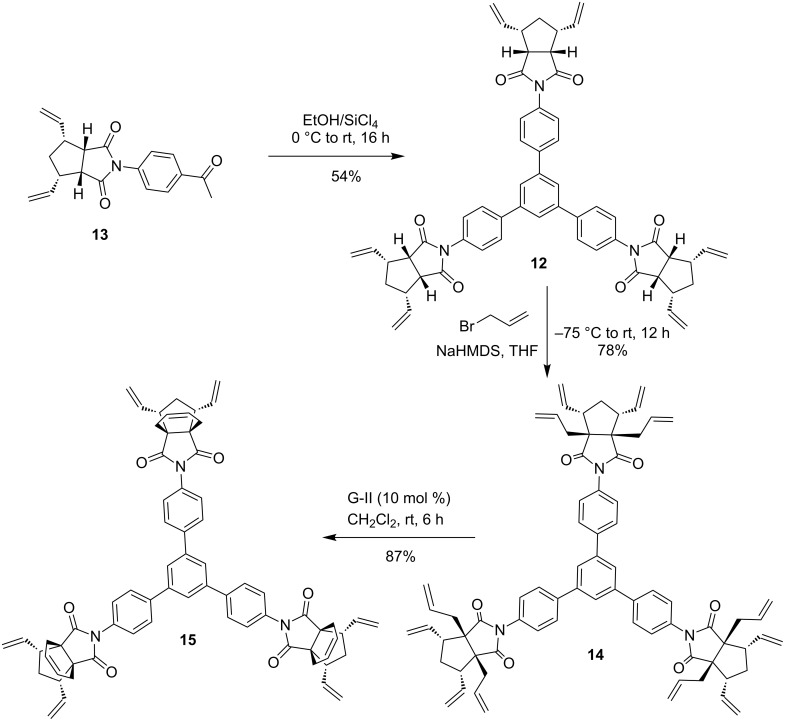
Synthesis of the *C*_3_-symmetric molecule **15** bearing propellane moieties via trimerization and RCM.

Having the ROM product **13** in hand, it was subjected to trimerization in the presence of EtOH/SiCl_4_ at 0 °C to room temperature to afford the trimerized product **12** in 54% yield. Next, the *C*_3_-symmetric product **12** was reacted with allyl bromide in the presence of sodium bis(trimethylsilyl)amide (NaHMDS, 1 M solution in THF) at −75 °C to deliver the RCM precursor **14** in good yield (78%). The hexaallyl derivative **14** was subjected to RCM in the presence of Grubbs second generation (G-II) catalyst in CH_2_Cl_2_ under nitrogen to give the propellane moiety bearing *C*_3_-symmetric product **15** in good yield (87%). Its structure was established on the basis of NMR spectral data, and its molecular formula was confirmed by HRMS data (Scheme 2).

**Scheme 3:**
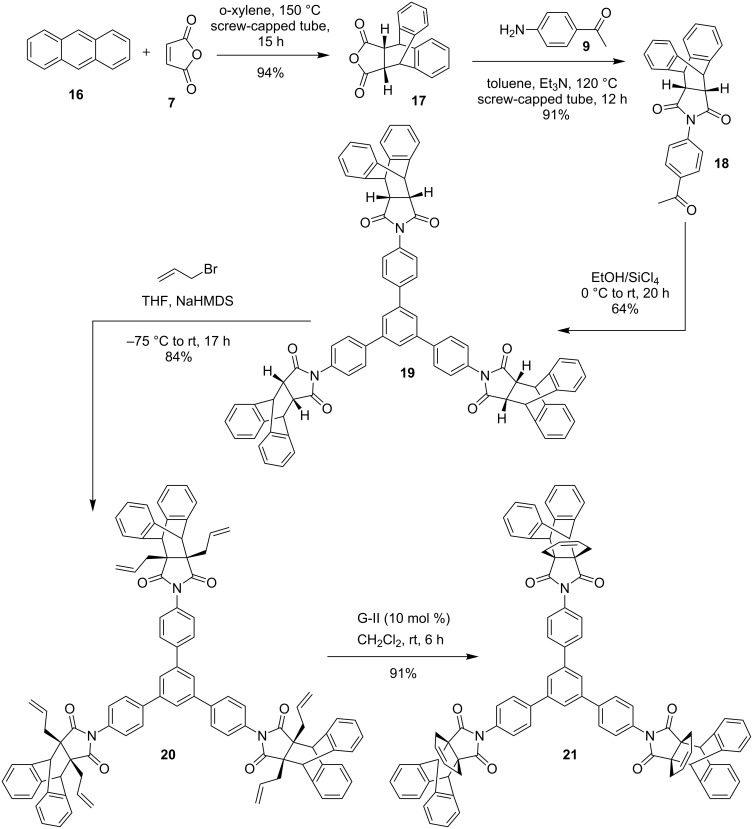
Synthesis of *C*_3_-symmetric molecule **21** bearing propellane moieties via trimerization and RCM.

Along similar lines, we expanded the scope of this strategy. To this end, commercially available anthracene (**16**) was reacted with maleic anhydride (**7**) in a screw-capped tube at 150 °C in *o*-xylene to obtain the DA adduct **17** in 94% yield [44–45]. Later, the DA adduct **17** was treated with 4-aminoacetophenone (**9**) in the presence of Et_3_N in toluene at 140 ºC to deliver the acetophenone derivative **18** (91% yield) and it was subjected to trimerization in the presence of EtOH/SiCl_4_ at 0 °C to rt to obtain the trimerized product **19** in 64% yield. Afterwards, the trimerized product **19** was treated with allyl bromide to accomplish *C*-allylation in the presence of NaHMDS (1 M solution in THF) at −75 °C to deliver the hexaallyl derivative **20** in 84% yield. Then, RCM in the presence of G-II catalyst in CH_2_Cl_2_ under nitrogen atmosphere gave the propellane moieties bearing *C*_3_-symmetric product **21** in good yield (91%). Its structure was established with the help of ^1^H NMR, ^13^C NMR spectral data and was further supported by HRMS details (Scheme 3).

## Conclusion

We have demonstrated a simple synthetic methodology to *C*_3_-symmetric star-shaped molecules containing propellane moieties at the periphery which may be useful for material science applications. Here, we have prepared DA adducts **8** and **17** from commercially available maleic anhydride (**7**), which was further utilized for trimerization and RCM sequence. We have successfully synthesized *C*_3_-symmetric molecules **15** and **21** bearing propellane moieties by employing RCM in the presence of 2nd generation (G-II) catalyst.

## Experimental

### General information

Some of these reactions were carried out in screw-capped tubes and other reactions under nitrogen or argon and ethylene atmosphere in oven-dried glassware. Air- and moisture-sensitive reactions were performed in degassed solvents. Transfer of moisture-sensitive materials were carried out using standard syringe−septum techniques. All the commercial grade reagents were used without any purification until otherwise specified. Melting points were recorded on a Veego or Büchi melting point apparatus and are uncorrected. NMR Spectra were generally recorded on Bruker (Avance 400 or Avance III 500) spectrometers operated at 400 or 500 MHz for ^1^H and 100 or 125.7 MHz for ^13^C nuclei. NMR Samples were generally made in chloroform-*d* solvent, and chemical shifts (δ values) are reported in parts per million (ppm). Coupling constants (*J* values) were reported in hertz (Hz). HRMS measurements were carried out using a Bruker (Maxis Impact) spectrometer. IR spectra were recorded on a Nicolet Impact-400 or Cary 630 FTIR spectrometer.

#### Synthesis of norbornene-based trimerized product **11**

To a solution of norbornene derivative **10** (500 mg, 1.77 mmol) in EtOH (8 mL), silicon tetrachloride (SiCl_4_, 0.61 mL, 5.36 mmol) was added dropwise at 0 °C and the reaction mixture was stirred for 10–15 min at the same temperature. Later, the reaction mixture was stirred at room temperature for 20 h. After completion of the reaction (TLC monitoring), the reaction mixture was quenched with sat. aq NH_4_Cl. Thereafter, the reaction mixture was diluted with EtOAc (10 mL) washed with water and brine (2 × 10 mL). Then, the aqueous layer was extracted with EtOAc (3 × 10 mL) and the combined organic layers were dried over Na_2_SO_4_. The solvent was removed under reduced pressure and the crude product was purified by silica gel column chromatography (65% EtOAc/petroleum ether) to afford the trimerized product **11** (321 mg, 64%) as a colourless solid. *R*_f_ = 0.54 (6:4 EtOAc/petroleum ether); mp 203–206 °C; ^1^H NMR (400 MHz, CDCl_3_) δ 7.68 (d, *J* = 5.2 Hz, 6H), 7.66 (s, 3H), 7.24 (d, *J* = 2.4 Hz, 6H), 6.28 (s, 6H), 3.51–3.44 (m, 12H), 1.79 (d, *J* = 8.8 Hz, 3H), 1.61 (d, *J* = 8.8 Hz, 3H) ppm; ^13^C NMR (125 MHz, CDCl_3_) δ 177.0, 141.8, 141.3, 134.8, 131.4, 128.2, 127.2, 125.7, 52.4, 46.0, 45.7 ppm; HRMS (ESI, Q-ToF) *m/z*: [M + H]^+^ calcd for C_51_H_40_N_3_O_6_, 790.2912; found, 790.2918; IR (neat) 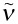
_max_: 2918, 1706, 1512, 1371, 1173, 754 cm^−1^.

#### Synthesis of ring open metathesis (ROM) product **13**

The solution of compound **10** (500 mg, 1.76 mmol) in dry CH_2_Cl_2_ (25 mL) was degasified by ethylene and G-I (5 mol %) was added to the reaction mixture at rt. Further, the reaction mixture was stirred for 48 h under ethylene atmosphere at rt. After completion of the reaction (TLC monitoring), the solvent was removed under reduced pressure. Later, the crude product was purified by silica gel column chromatography (30% EtOAc/petroleum ether) to obtain the ROM product **13** as a colourless solid (310 mg, 56%); *R*_f_ = 0.68 (4:6 EtOAc/petroleum ether); mp 143–145 °C; ^1^H NMR (400 MHz, CDCl_3_) δ 8.03 (d, *J* = 8.4 Hz, 2H), 7.40 (d, *J* = 8.4 Hz, 2H), 6.12–6.03 (m, 2H), 5.20–5.15 (m, 4H), 3.43 (q, *J* = 2.0 Hz, 2H), 3.08–3.00 (m, 2H), 2.60 (s, 3H), 2.08–2.02 (m, 1H), 1.57 (t, *J* = 6.4 Hz, 1H) ppm; ^13^C NMR (100 MHz, CDCl_3_) δ 197.2, 175.4, 136.7, 136.2, 136.1, 129.2, 126.5, 116.3, 49.1, 46.4, 35.4, 26.8 ppm; HRMS (ESI, Q-ToF) *m/z*: [M + Na]^+^ calcd for C_19_H_19_NO_3_·Na, 332.1257; found, 332.1254; IR (neat) 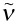
_max_: 2325, 1671, 1263, 746 cm^−1^.

#### Synthesis of trimerized compound **12**

Based on the earlier procedure of trimerization, compound **13** (500 mg, 1.61 mmol) was treated with SiCl_4_ (0.55 mL, 4.84 mmol) in the presence of EtOH (8 mL) for 16 h to afford trimerized product **12** after silica gel column chromatography (60% EtOAc/petroleum ether) as a colourless solid (254 mg, 54%); mp 152–154 °C; *R*_f_ = 0.55 (5:5 EtOAc/petroleum ether); ^1^H NMR (400 MHz, CDCl_3_) δ 7.72 (d, *J* = 8.0 Hz, 6H), 7.70 (s, 3H), 7.37 (d, *J* = 8.4 Hz, 6H), 6.15–6.07 (m, 6H), 5.19 (q, *J* = 8.4 Hz, 12H), 3.43 (dd, *J*_1_ = 2.0 Hz, *J*_2_ = 2.0 Hz, 6H), 3.07–2.99 (m, 6H), 2.04 (q, *J* = 6.8 Hz, 3H), 1.58 (q, *J* = 13.2 Hz, 3H) ppm; ^13^C NMR (100 MHz, CDCl_3_) δ 175.7, 141.7, 141.1, 136.4, 131.4, 128.1, 126.9, 125.6, 116.1, 49.1, 46.3, 35.5 ppm; HRMS (ESI, Q-ToF) *m/z*: [M + Na]^+^ calcd for C_57_H_51_N_3_O_6_·Na, 896.3670; found; 896.3678; IR (neat) 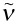
_max_: 2342, 1709, 1512, 1183, 919, 736 cm^−1^.

#### Synthesis of trimerized product **19**

Based on the earlier procedure of trimerization, compound **18** (500 mg, 1.27 mmol) was treated with SiCl_4_ (0.43 mL, 3.83 mmol) in the presence of EtOH (8 mL) for 20 h to give the trimerized product **19** after silica gel (100–200 mesh) column chromatography (50% EtOAc/petroleum ether) as a colourless solid (324 mg, 64%); *R*_f_ = 0.61 (4:6 EtOAc/petroleum ether); mp 224–226 °C; ^1^H NMR (500 MHz, CDCl_3_) δ 7.56 (s, 3H), 7.51 (d, *J* = 8.0 Hz, 6H), 7.43 (d, *J* = 3.0 Hz, 6H), 7.37 (d, *J* = 3.0 Hz, 6H), 7.24–7.22 (m, 12H), 6.62 (d, *J* = 8.5 Hz, 6H), 4.92 (s, 6H), 3.41 (s, 6H) ppm; ^13^C NMR (125 MHz, CDCl_3_) δ 176.3, 141.5, 141.4, 138.9, 128.2, 127.4, 127.1, 127.0, 125.3, 124.5, 47.2, 46.1 ppm; HRMS (ESI, Q-ToF) *m/z*: [M + Na]^+^ calcd for C_78_H_51_N_3_O_6_·Na, 1148.3670; found, 1148.3672; IR (neat) 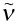
_max_: 2318, 1266, 745, 707 cm^−1^.

#### Synthesis of hexaallyl derivative **14**

To the solution of compound **12** (200 mg, 0.22 mmol) in anhydrous THF (15 mL) was added NaHMDS (2 mL of 1 M solution in THF, 1.93 mmol) at −75 °C and the reaction mixture was stirred for 30 min under nitrogen atmosphere. Then allyl bromide (0.11 mL, 1.60 mmol) was added to the reaction mixture and stirred for 2 h at −75 °C. Later, the reaction mixture was stirred to room temperature for 10 h. After completion of the reaction (TLC monitoring), the reaction mixture was quenched with 1 M aq HCl solution, and the aqueous layer was extracted by EtOAc (3 × 10 mL). Then the organic fraction was washed with brine solution, dried over Na_2_SO_4_ and concentrated. The crude residue was purified by silica gel column chromatography (10% EtOAc/petroleum ether) to afford hexa-allyl derivative **14** as a colourless solid (199 mg, 78%). *R*_f_ = 0.60 (3:7 EtOAc/petroleum ether); mp 204–206 °C; ^1^H NMR (500 MHz, CDCl_3_) δ 7.71 (d, *J* = 5.5 Hz, 6H), 7.69 (s, 3H), 7.33 (d, *J* = 8.5 Hz, 6H), 6.08–5.96 (m, 12H), 5.28–5.13 (m, 24H), 2.77–2.66 (m, 18H), 2.04–2.00 (m, 3H), 1.65 (q, *J* = 12.5 Hz, 3H) ppm; ^13^C NMR (125 MHz, CDCl_3_) δ 178.2, 141.9, 141.3, 136.5, 132.8, 131.5, 128.1, 127.1, 125.7, 120.3, 117.1, 59.9, 51.2, 36.6, 35.1 ppm; HRMS (ESI, Q-ToF) *m/z*: [M + Na]^+^ calcd for C_75_H_75_N_3_O_6_·Na, 1136.5548; found, 1136.5544; IR (neat) 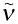
_max_: 2345, 1671, 1263, 746 cm^−1^.

#### Synthesis of hexaallyl product **20**

Based on the earlier procedure of allylation, compound **19** (336 mg, 0.29 mmol) was treated with NaHMDS (2.3 mL of 1 M solution in THF, 2.39 mmol) and allyl bromide (0.14 mL, 1.93 mmol) for 17 h to deliver hexaallyl product **20** after silica gel column chromatography (20% EtOAc/petroleum ether) as a colourless solid (345 mg, 84%); *R*_f_ = 0.83 (2:8 EtOAc/petroleum ether); mp 195–197 °C; ^1^H NMR (400 MHz, CDCl_3_) δ 7.57 (s, 3H), 7.51 (d, *J* = 8.8 Hz, 6H), 7.40 (q, *J* = 3.2 Hz, 6H), 7.32 (q, *J* = 3.2 Hz, 6H), 7.24–7.20 (m, 12H), 6.58 (d, *J* = 8.0 Hz, 6H), 6.33–6.23 (m, 6H), 5.20 (dd, *J*_1_ = 11.6 Hz, *J*_2_ = 17.2 Hz, 12H), 4.68 (s, 6H), 2.45 (dd, *J*_1_ = 5.6 Hz, *J*_2_ = 5.6 Hz, 6H), 2.16 (q, *J* = 8.8 Hz, 6H) ppm; ^13^C NMR (100 MHz, CDCl_3_) δ 178.5, 141.7, 141.4, 139.9, 139.4, 133.6, 131.0, 128.1, 127.2, 127.1, 126.7, 126.5, 125.6, 125.3, 119.4, 55.7, 51.6, 37.3 ppm; HRMS (ESI, Q-ToF) *m/z*: [M + Na]^+^ calcd for C_96_H_75_N_3_O_6_·Na, 1388.5548; found, 1389.5585; IR (neat) 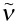
_max_: 2925, 2335, 1706, 1461, 1376, 1273, 741 cm^–1^.

### General procedure for ring-closing metathesis (RCM)

The solution of hexaallyl derivatives **14** or **20** in dry CH_2_Cl_2_ (20 mL) was degassed by nitrogen and G-II (10 mol %) was added to the reaction mixture. Further, the reaction mixture was stirred for 6 h under nitrogen atmosphere at room temperature. After completion of the reaction (TLC monitoring), the solvent was removed under reduced pressure. The crude product was purified by silica gel column chromatography (EtOAc/petroleum ether) to afford the propellane bearing *C*_3_-symmetric products **15** or **21**.

#### Synthesis of RCM derivative **15**

Colourless solid, 87% (121 mg, starting with 150 mg of hexaallyl compound **14**); *R*_f_ = 0.60 (3:7 EtOAc/petroleum ether); mp 272–275 °C; ^1^H NMR (400 MHz, CDCl_3_) δ 7.71 (d, *J* = 2.0 Hz, 6H), 7.69 (s, 3H), 7.35 (d, *J* = 8.4 Hz, 6H), 6.08–5.99 (m, 12H), 5.18–5.14 (m, 12H), 2.76 (dd, *J*_1_ = 4.0 Hz, *J*_2_ = 3.2 Hz, 6H), 2.67–2.61 (m, 6H), 2.23 (dd, *J*_1_ = 2.0 Hz, *J*_2_ = 2.0 Hz, 6H), 2.04–1.98 (m, 3H), 1.58 (q, *J* = 12.8, 3H) ppm; ^13^C NMR (100 MHz, CDCl_3_) δ 178.2, 141.8, 141.2, 136.2, 131.6, 128.5, 128.1, 126.9, 125.7, 116.4, 58.7, 53.3, 35.7, 30.9 ppm; HRMS (ESI, Q-ToF) *m/z*: [M + Na]^+^ calcd for C_69_H_63_N_3_O_6_·Na, 1052.4609; found, 1052.4617; IR (neat) 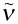
_max_: 2305, 1651, 1363, 844 cm^−1^.

#### Synthesis of RCM derivative **21**

Colourless solid, 91% (258 mg, starting with 300 mg of hexaallyl product **20**); *R*_f_ = 0.75 (3:7 EtOAc/petroleum ether); mp 264–267 °C; ^1^H NMR (400 MHz, CDCl_3_) δ 7.56 (s, 3H), 7.49 (d, *J* = 6.8 Hz, 6H), 7.43 (d, *J* = 2.4 Hz, 6H), 7.34 (d, *J* = 2.4 Hz, 6H), 7.25–7.23 (m, 12H), 6.57 (d, *J* = 7.6 Hz, 6H), 5.83 (s, 6H), 4.47 (s, 6H), 2.90 (d, *J* = 14.4 Hz, 6H), 1.80 (d, *J* = 14.8 Hz, 6H) ppm; ^13^C NMR (100 MHz, CDCl_3_) δ 180.0, 141.6, 141.4, 140.4, 140.2, 131.2, 128.0, 127.9, 127.2, 127.1, 127.0, 126.5, 125.6, 125.2, 57.4, 51.5, 30.2 ppm; HRMS (ESI, Q-ToF) *m/z*: [M + K]^+^ calcd for C_90_H_63_N_3_O_6_·K, 1320.4348; found, 1320.4344; IR (neat) 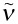
_max_: 2328, 1708, 1383, 837, 690 cm^−1^.

## Supporting Information

File 1 Copies of ^1^H, ^13^C NMR and HRMS spectra of new compounds.
