# Supplementary material for: Design and synthesis of C3-symmetric molecules bearing propellane moieties via cyclotrimerization and a ring-closing metathesis sequence
Source: Beilstein J Org Chem. 2018 Oct 1;14:2537–44. doi: 10.3762/bjoc.14.230 (PMC6204777; doi:10.3762/bjoc.14.230)

Supporting Information

for

Design and synthesis of C_3-symmetric molecules bearing propellane moieties via cyclotrimerization and a ring-closing metathesis sequence

Sambasivarao Kotha*, Saidulu Todeti, and Vikas R. Aswar

Address: Department of Chemistry, Indian Institute of Technology Bombay, Powai, Mumbai-400076, India, Fax: +91(22)-2572 7152

Email: Sambasivarao Kotha - srk@chem.iitb.ac.in

*Corresponding author

Copies of ^{1}H, ^{13}C NMR and HRMS spectra of new compounds

^{1}H NMR of compound 11 in $CDCl_3$

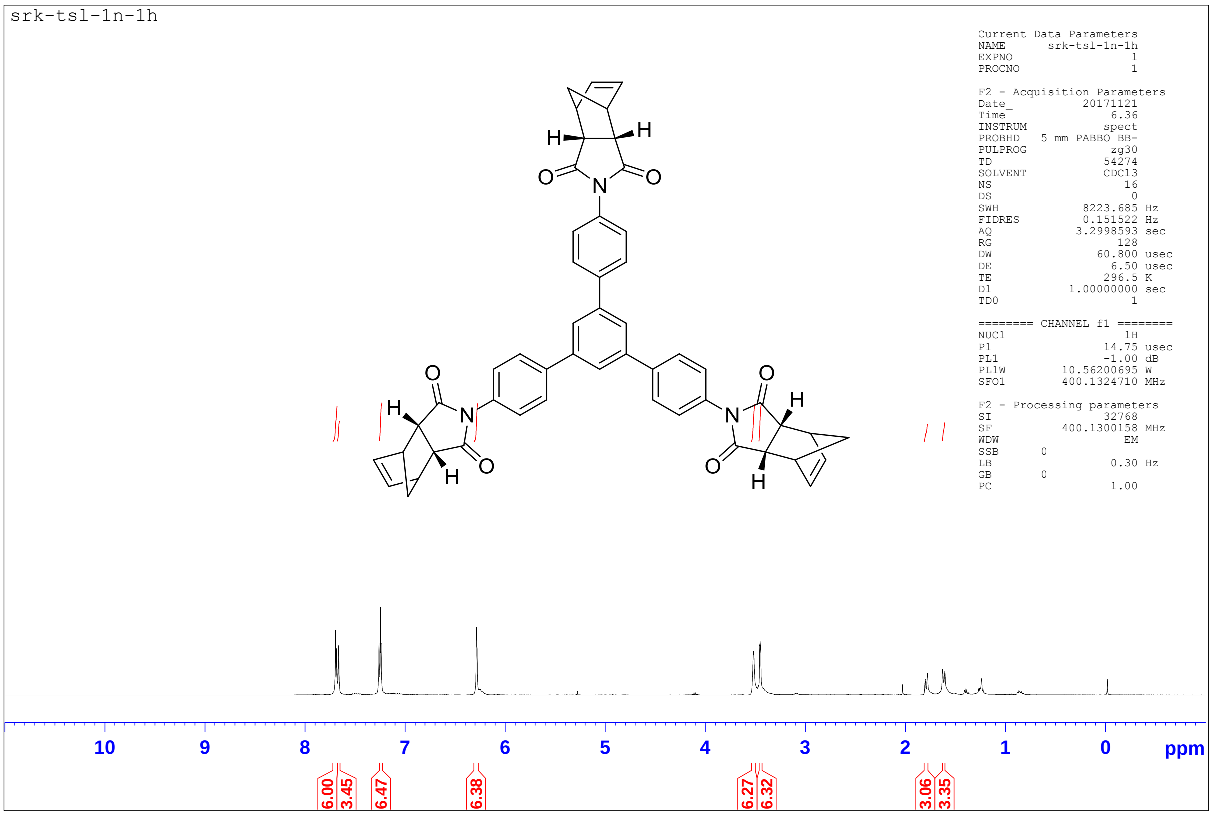

^{13}C NMR of compound 11 in $CDCl_3$

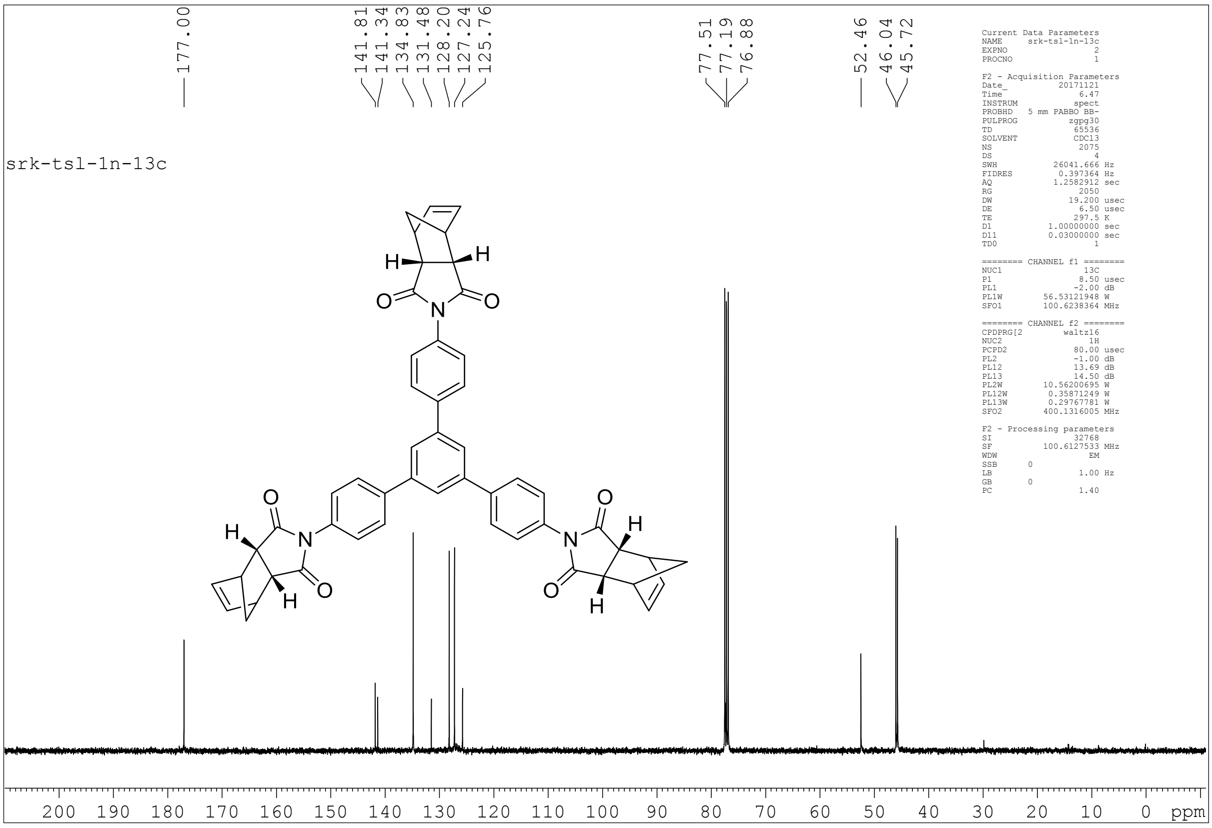

HRMS of Compound 11

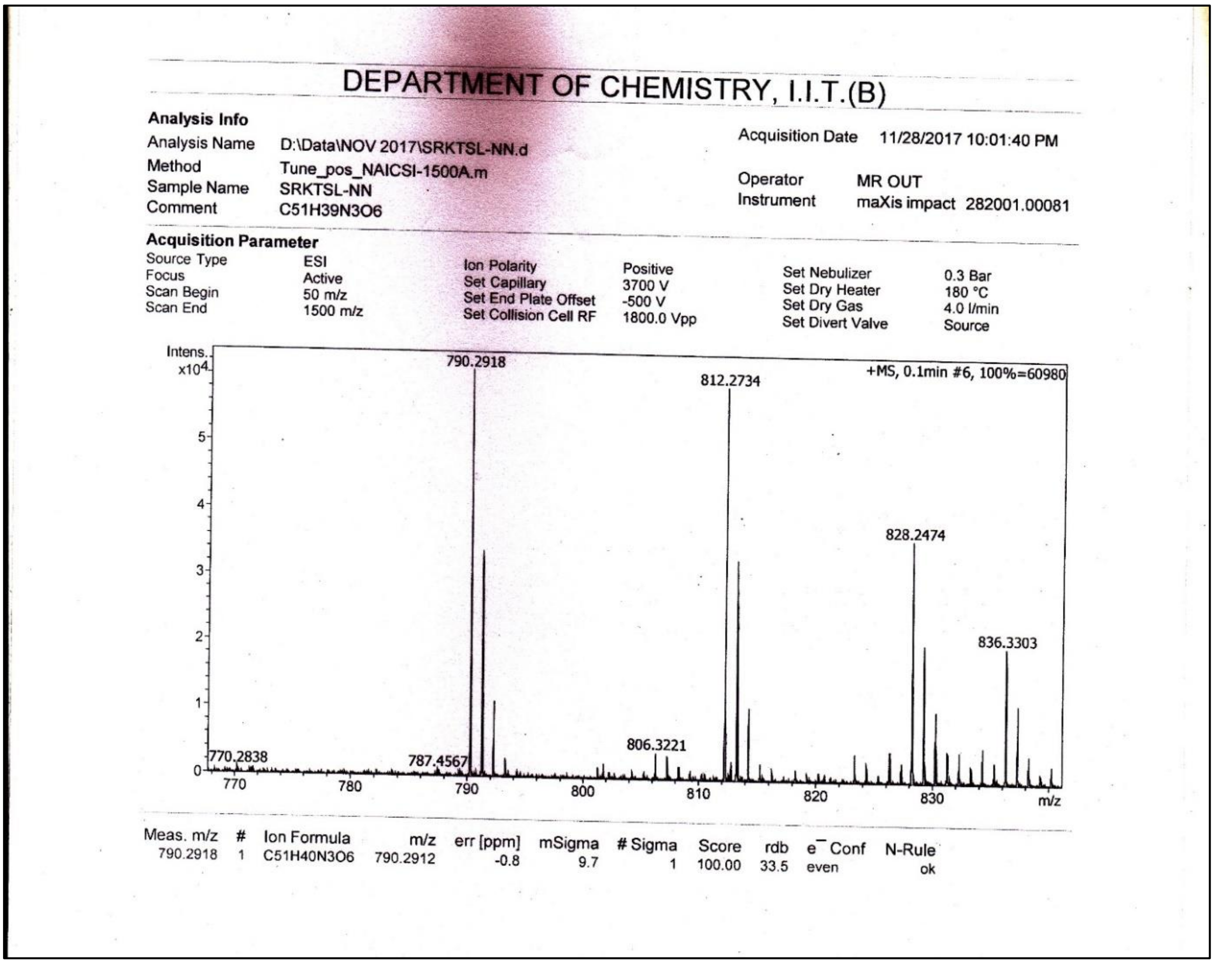
DEPARTMENT OF CHEMISTRY, I.I.T.(B)

Analysis Info

Analysis Name	D:\Data\NOV 2017\SRKTSL-NN.d	Acquisition Date	11/28/2017 10:01:40 PM
Method	Tune_pos_NAICSI-1500A.m	Operator	MR OUT
Sample Name	SRKTSL-NN	Instrument	maXis impact 282001.00081
Comment	C51H39N3O6		

Acquisition Parameter

Source Type	ESI	Ion Polarity	Positive	Set Nebulizer	0.3 Bar
Focus	Active	Set Capillary	3700 V	Set Dry Heater	180 °C
Scan Begin	50 m/z	Set End Plate Offset	-500 V	Set Dry Gas	4.0 l/min
Scan End	1500 m/z	Set Collision Cell RF	1800.0 Vpp	Set Divert Valve	Source

Meas. m/z	#	Ion Formula	m/z	err [ppm]	mSigma	# Sigma	Score	rdb	e⁻ Conf	N-Rule
790.2918	1	C51H40N3O6	790.2912	-0.8	9.7	1	100.00	33.5	even	ok

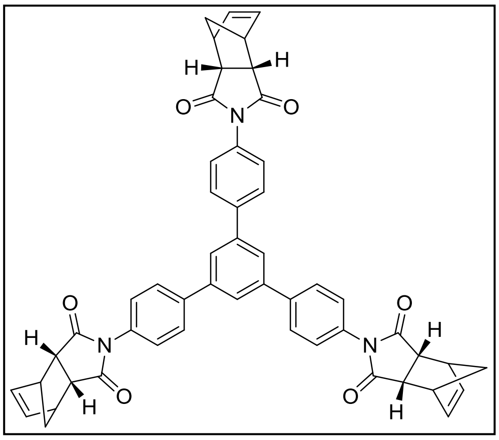

^{1}H NMR of compound 19 in $CDCl_3$

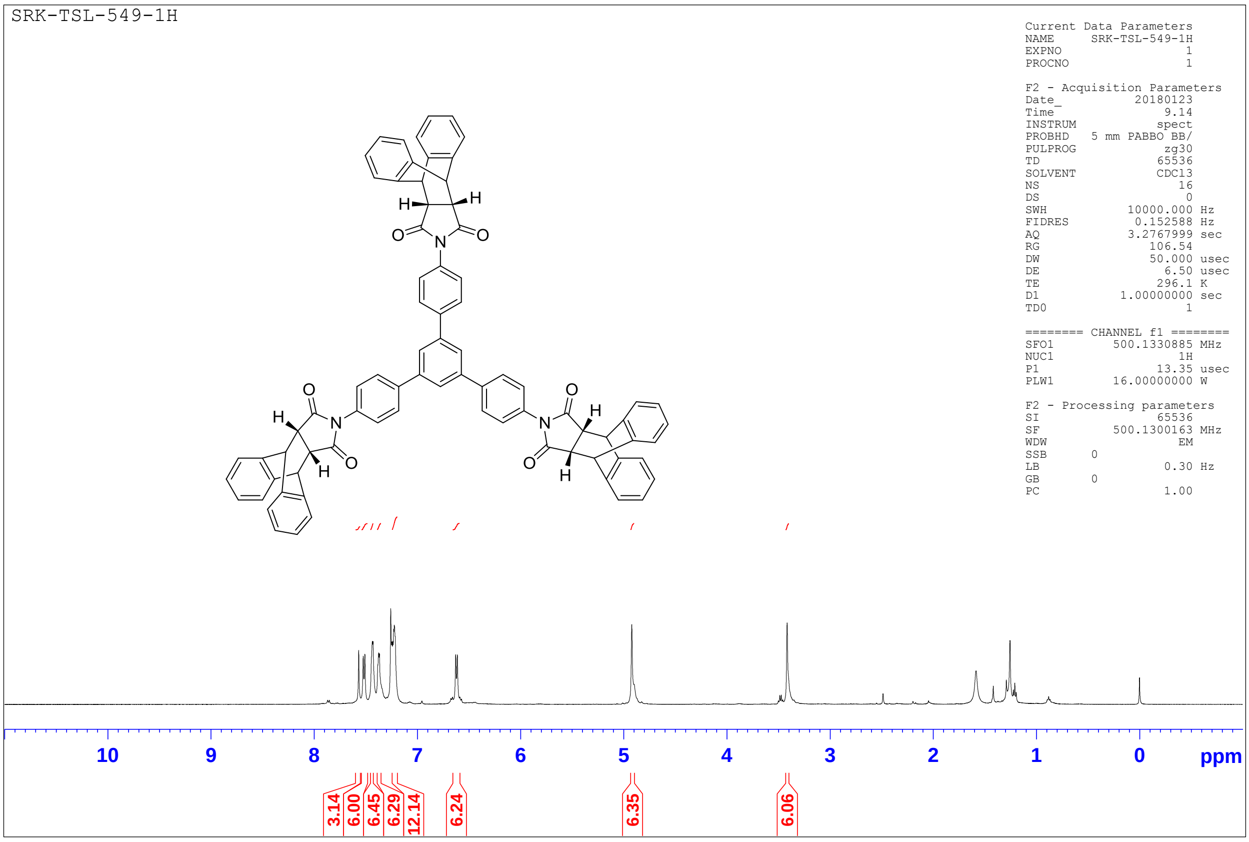

^{13}C NMR of compound 19 in $CDCl_3$

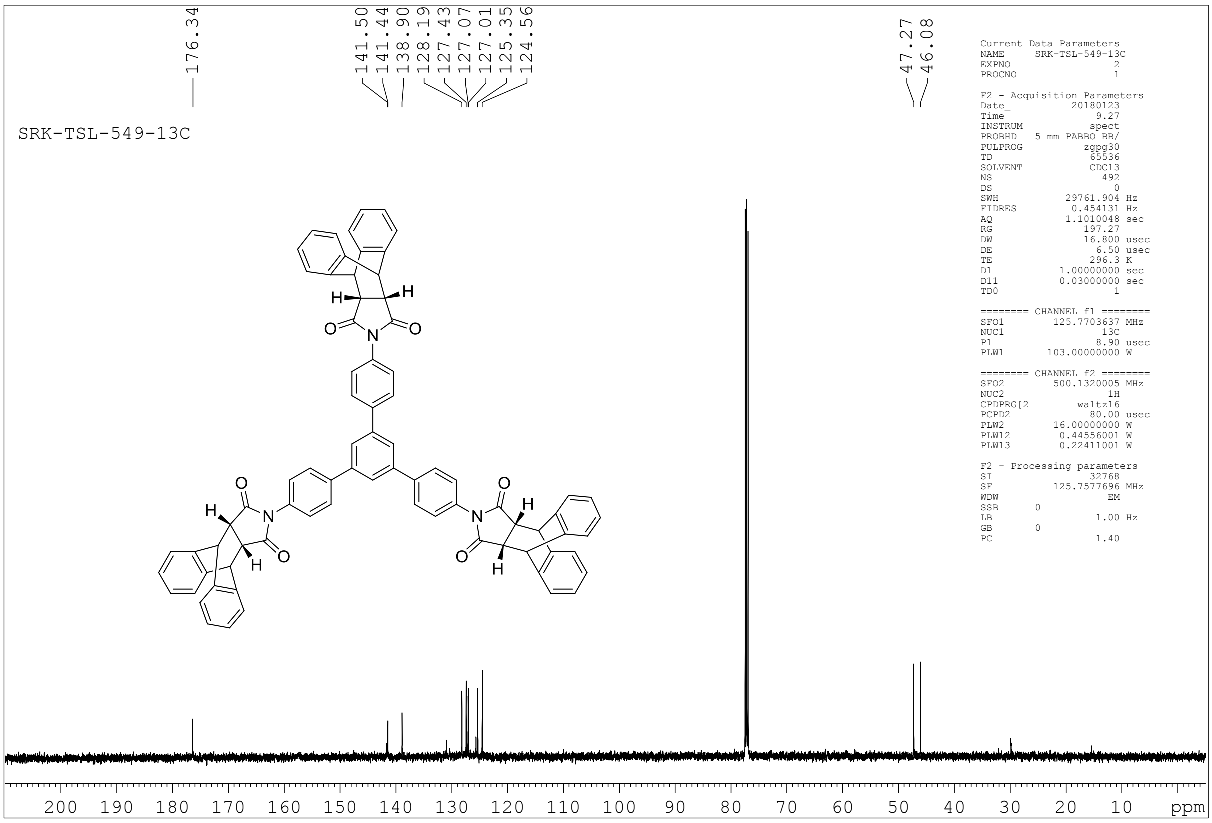

HRMS of Compound 19

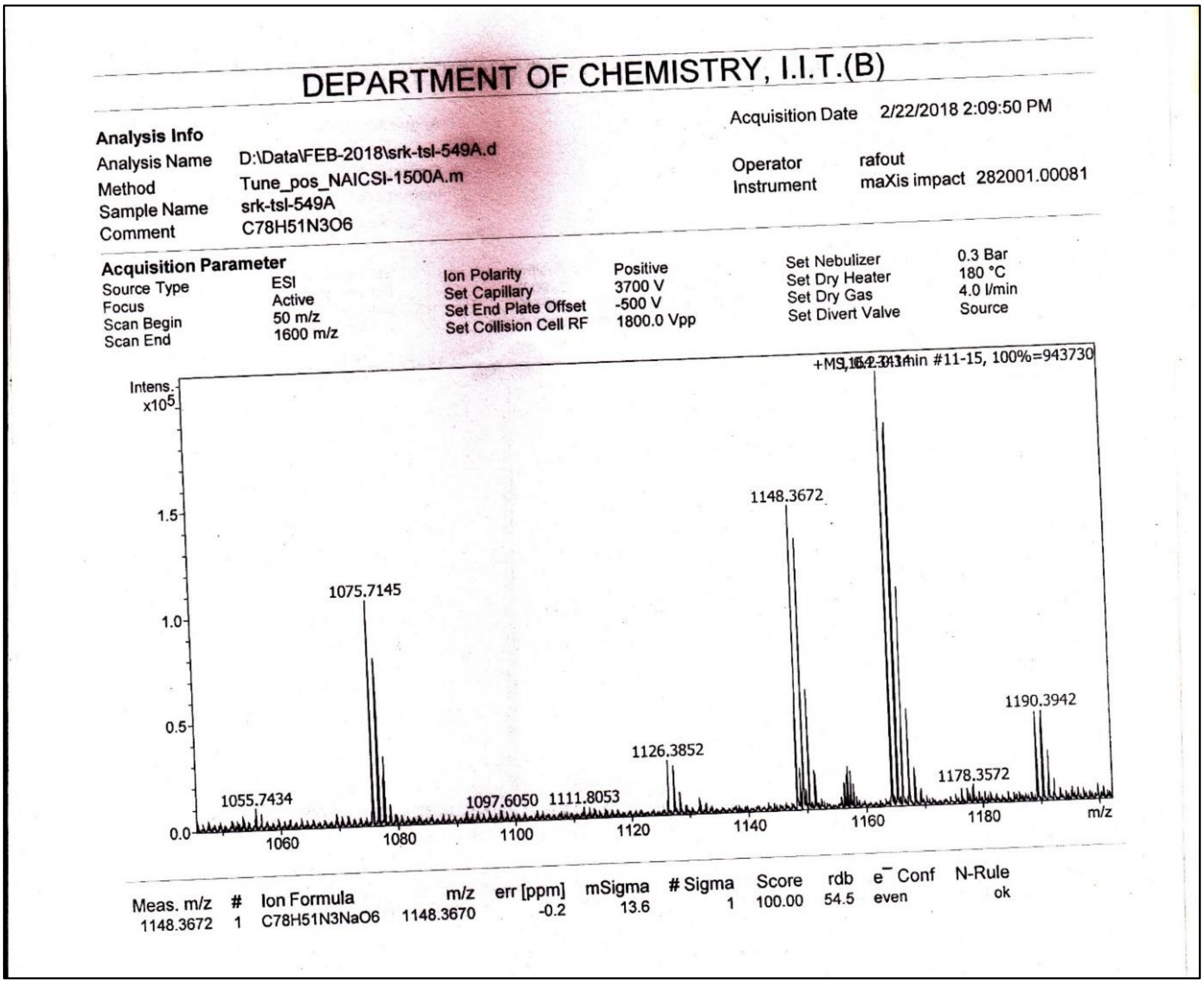

DEPARTMENT OF CHEMISTRY, I.I.T.(B)

Analysis Info

Analysis Name D:\Data\FEB-2018\srk-tsl-549A.d
Method Tune_pos_NAICSI-1500A.m
Sample Name srk-tsl-549A
Comment C78H51N3O6

Acquisition Date 2/22/2018 2:09:50 PM
Operator rafout
Instrument maXis impact 282001.00081

Acquisition Parameter

Source Type	ESI	Ion Polarity	Positive	Set Nebulizer	0.3 Bar
Focus	Active	Set Capillary	3700 V	Set Dry Heater	180 °C
Scan Begin	50 m/z	Set End Plate Offset	-500 V	Set Dry Gas	4.0 l/min
Scan End	1600 m/z	Set Collision Cell RF	1800.0 Vpp	Set Divert Valve	Source

Meas. m/z	#	Ion Formula	m/z	err [ppm]	mSigma	# Sigma	Score	rdb	e⁻ Conf	N-Rule
1148.3672	1	C78H51N3NaO6	1148.3670	-0.2	13.6	1	100.00	54.5	even	ok

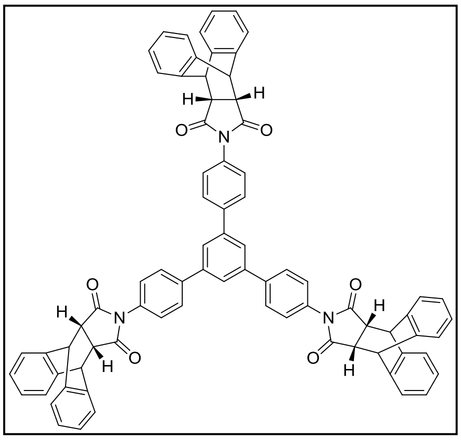

^{1}H NMR of compound 20 in $CDCl_3$

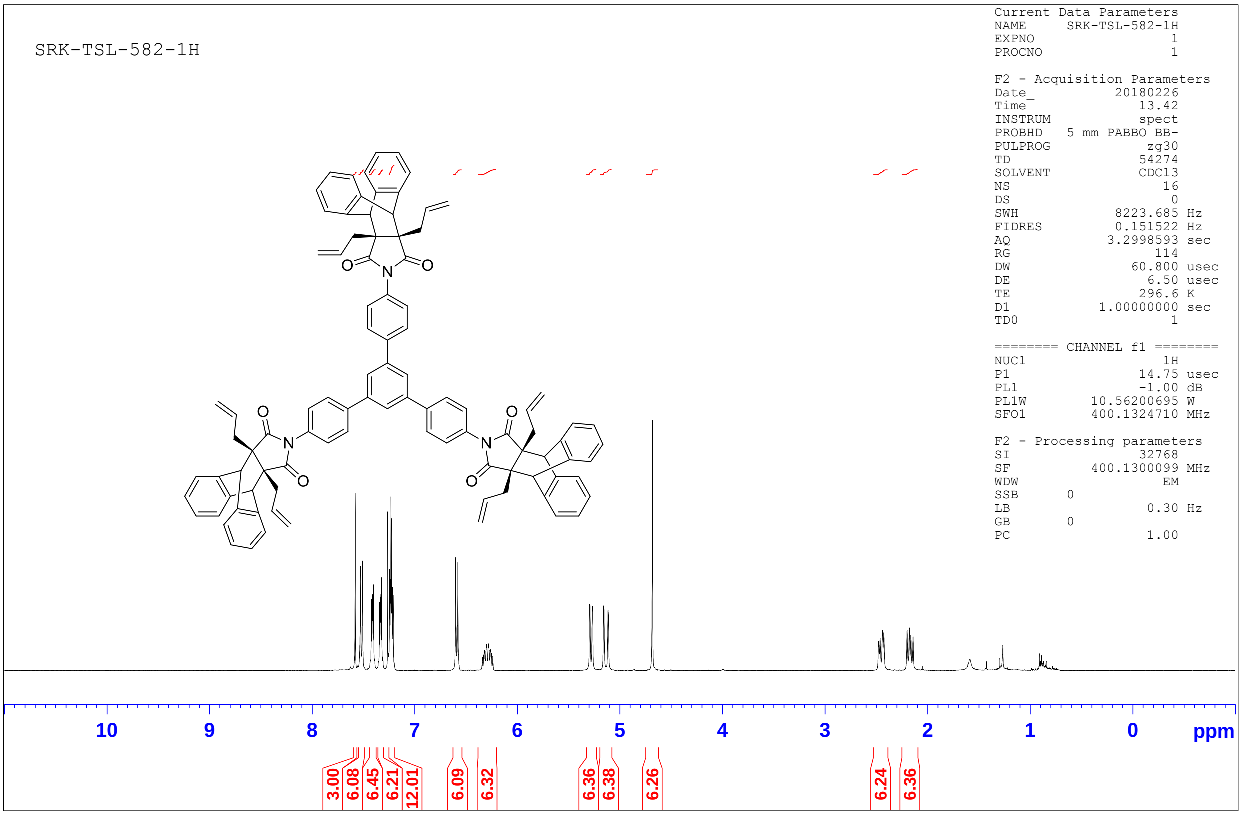

^{13}C NMR of compound 20 in $CDCl_3$

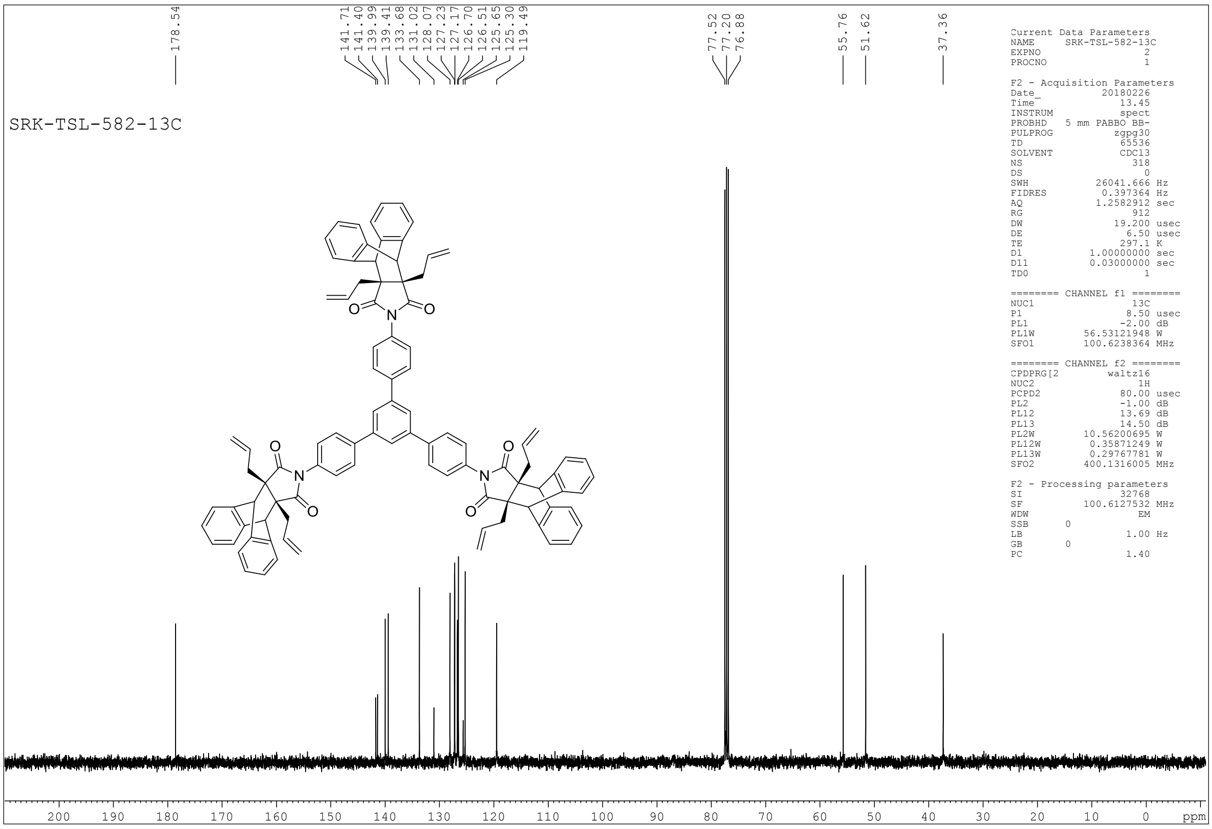

HRMS of compound 20

DEPARTMENT OF CHEMISTRY, I.I.T.(B)

Analysis Info

Analysis Name	D:\Data\FEB-2018\SRK-TSL-570A.d	Acquisition Date	2/22/2018 3:20:23 PM
Method	Tune_pos_NAICSI-2500.m	Operator	rafout
Sample Name	SRK-TSL-570A	Instrument	maXis impact 282001.00081
Comment	C96H75N3O6		

Acquisition Parameter

Source Type	ESI	Ion Polarity	Positive	Set Nebulizer	0.3 Bar
Focus	Active	Set Capillary	3700 V	Set Dry Heater	180 °C
Scan Begin	50 m/z	Set End Plate Offset	-500 V	Set Dry Gas	4.0 l/min
Scan End	2500 m/z	Set Collision Cell RF	2100.0 Vpp	Set Divert Valve	Source

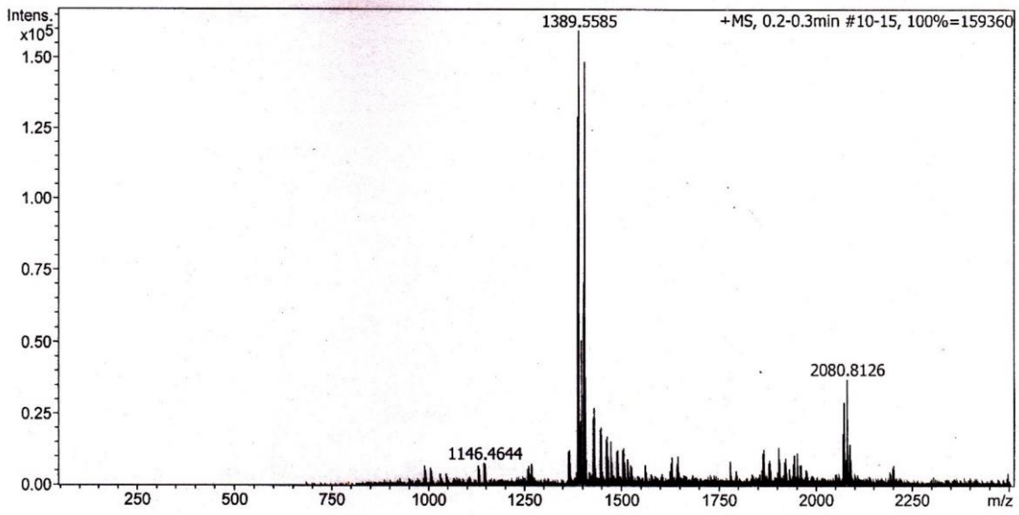

Meas. m/z	#	Ion Formula	m/z	err [ppm]	mSigma	# Sigma	Score	rdb	e⁻ Conf	N-Rule
1389.5585	1	C96H75N3NaO6	1388.5548	0.3	59.1	1	100.00	60.5	even	ok

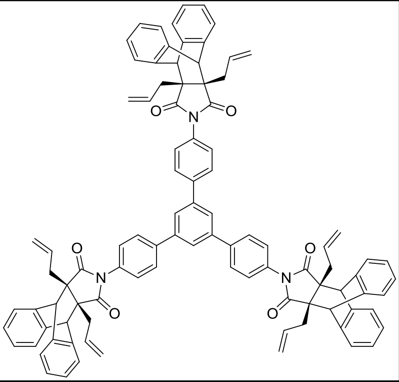

^{1}H NMR of compound 13 in $CDCl_3$

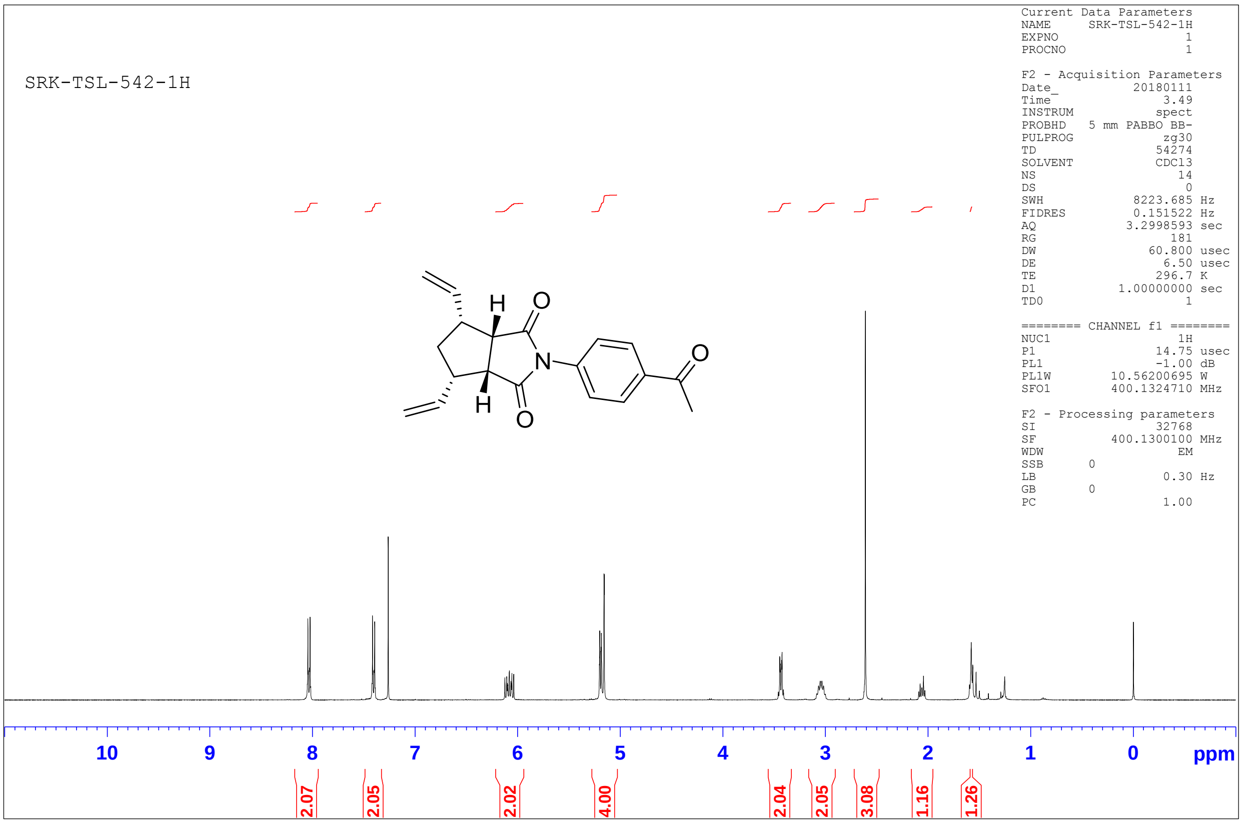

^{13}C NMR of compound 13 in $CDCl_3$

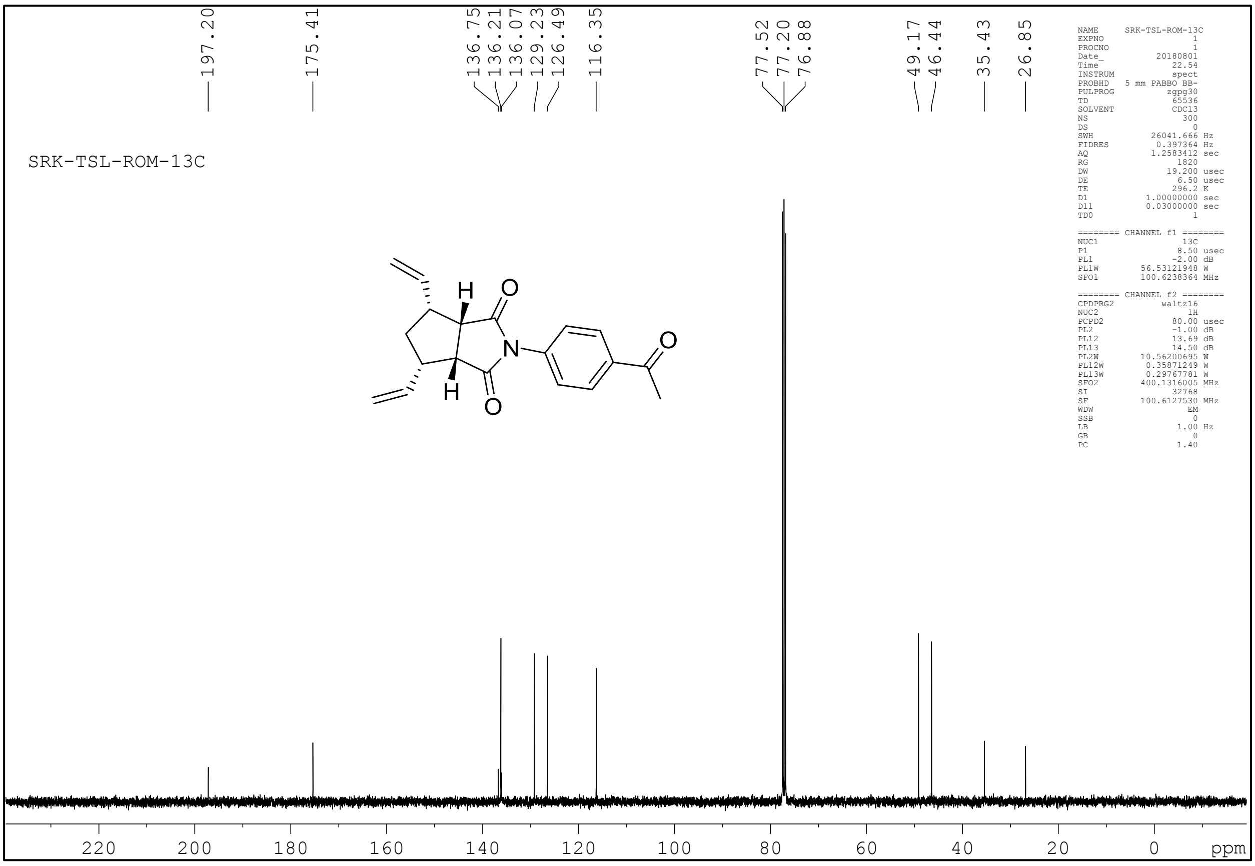

HRMS of Compound 13

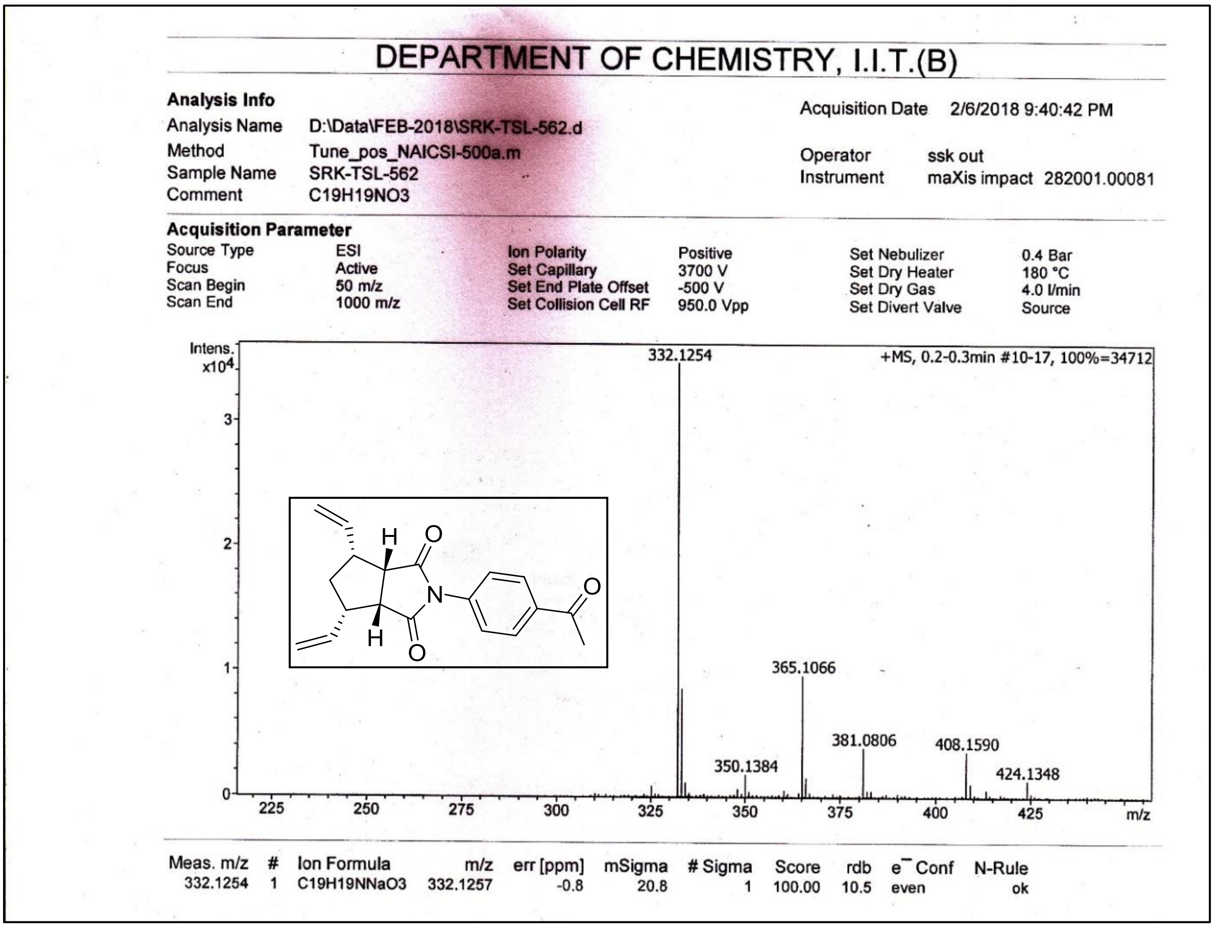

DEPARTMENT OF CHEMISTRY, I.I.T.(B)

Analysis Info

Analysis Name	D:\Data\FEB-2018\SRK-TSL-562.d	Acquisition Date	2/6/2018 9:40:42 PM
Method	Tune_pos_NAICSI-500a.m	Operator	ssk out
Sample Name	SRK-TSL-562	Instrument	maXis impact 282001.00081
Comment	C19H19NO3		

Acquisition Parameter

Source Type	ESI	Ion Polarity	Positive	Set Nebulizer	0.4 Bar
Focus	Active	Set Capillary	3700 V	Set Dry Heater	180 °C
Scan Begin	50 m/z	Set End Plate Offset	-500 V	Set Dry Gas	4.0 l/min
Scan End	1000 m/z	Set Collision Cell RF	950.0 Vpp	Set Divert Valve	Source

Meas. m/z	#	Ion Formula	m/z	err [ppm]	mSigma	# Sigma	Score	rdb	e⁻ Conf	N-Rule
332.1254	1	C19H19NNaO3	332.1257	-0.8	20.8	1	100.00	10.5	even	ok

^{1}H NMR of compound 12 in $CDCl_3$

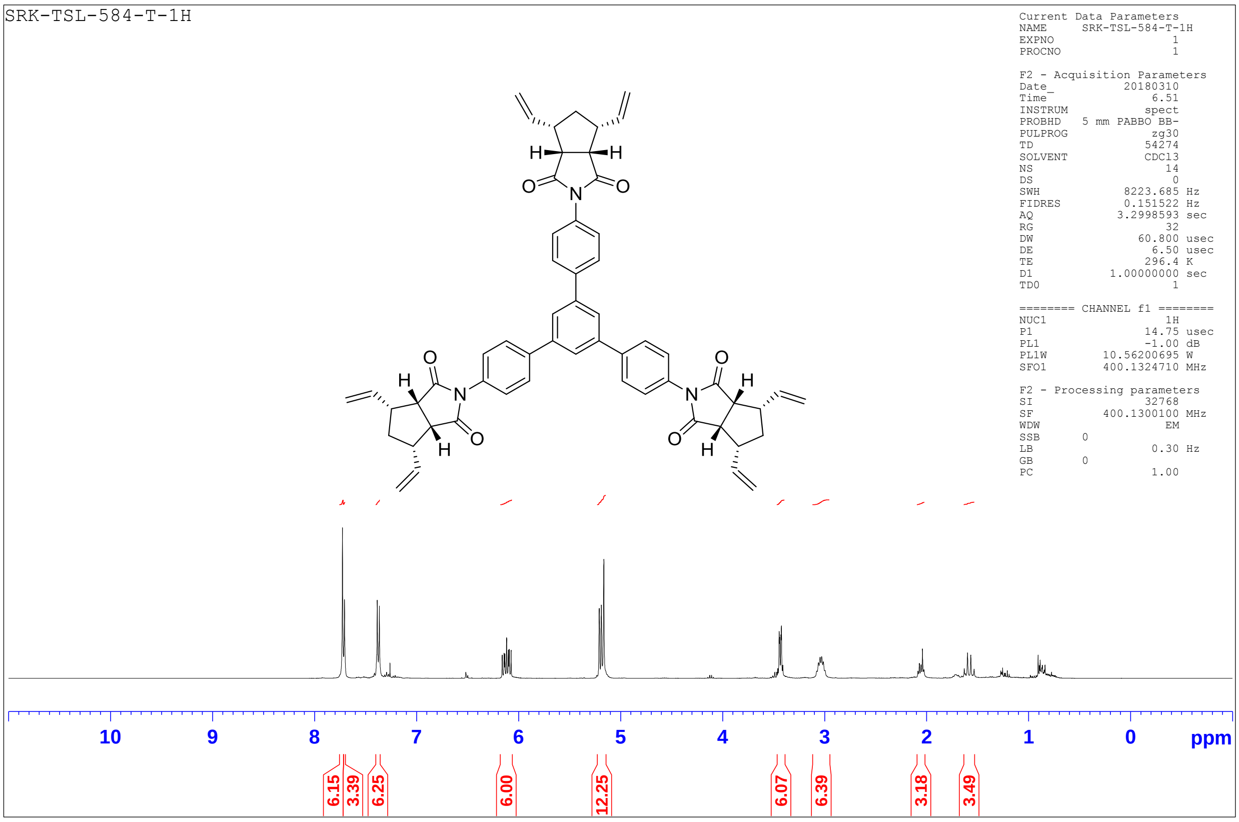

^{13}C NMR of compound 12 in $CDCl_3$

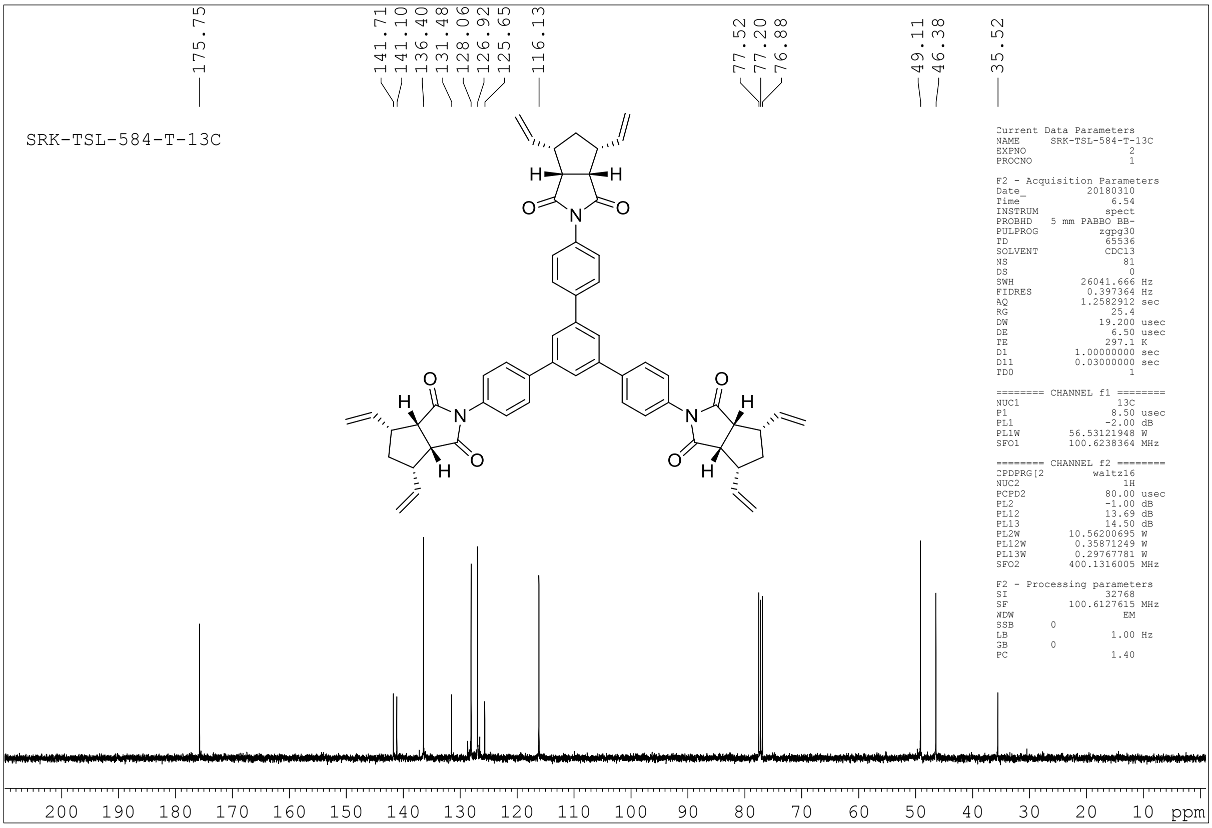

HRMS of Compound 12

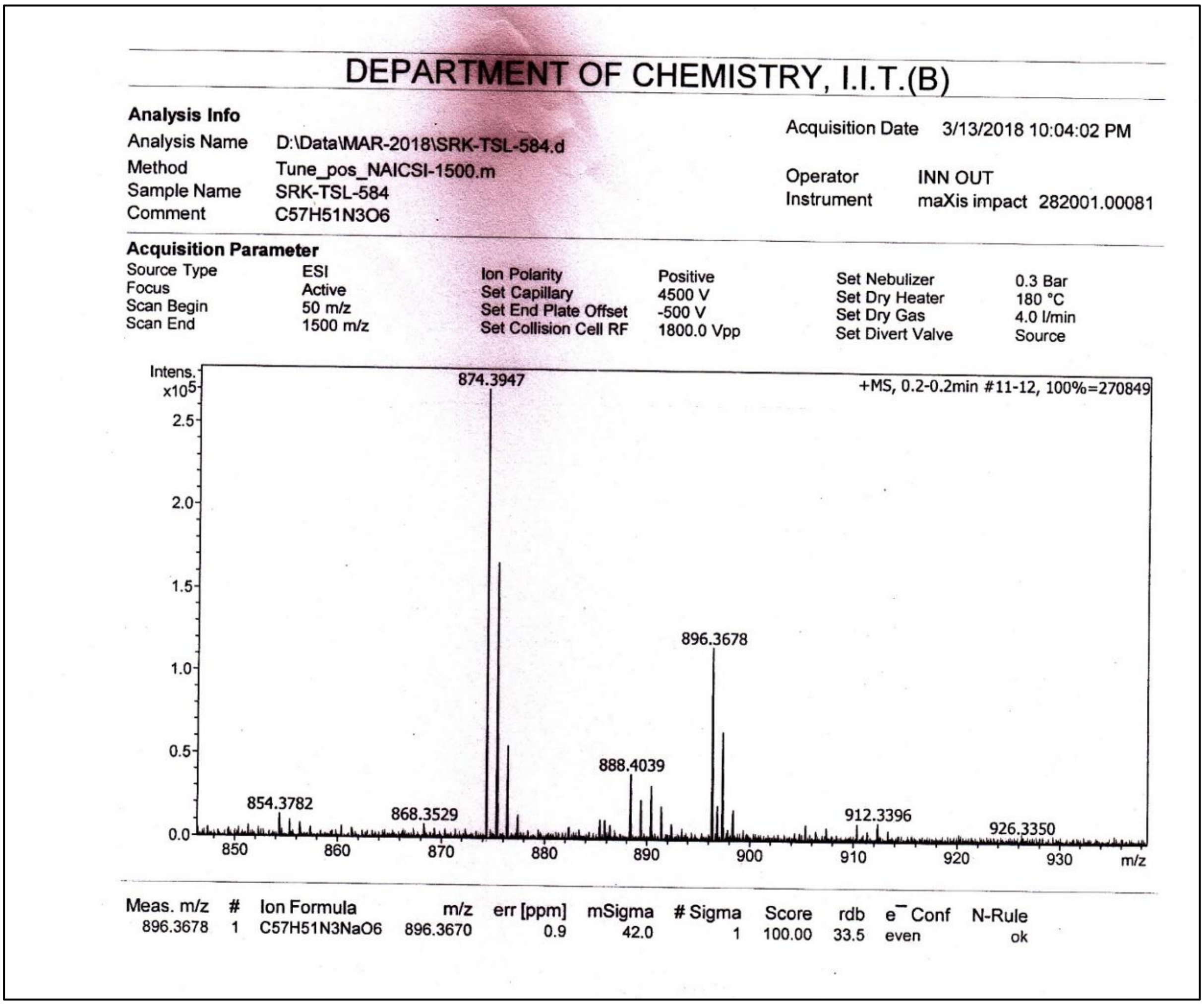

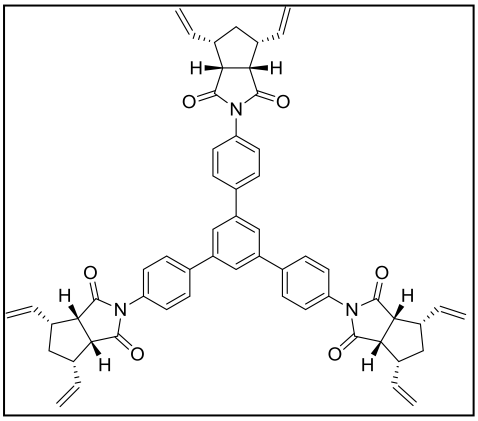

^{1}H NMR of compound 14 in $CDCl_3$

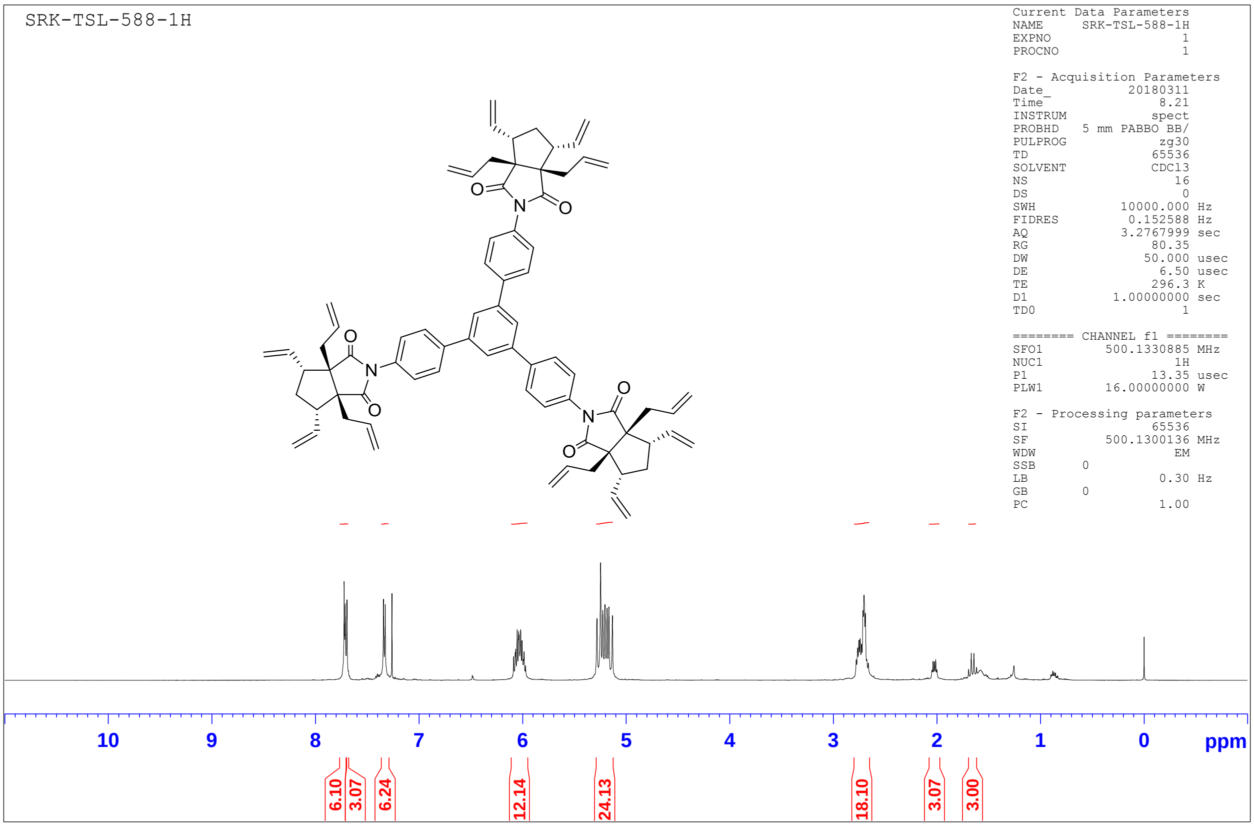

^{13}C NMR of compound 14 in $CDCl_3$

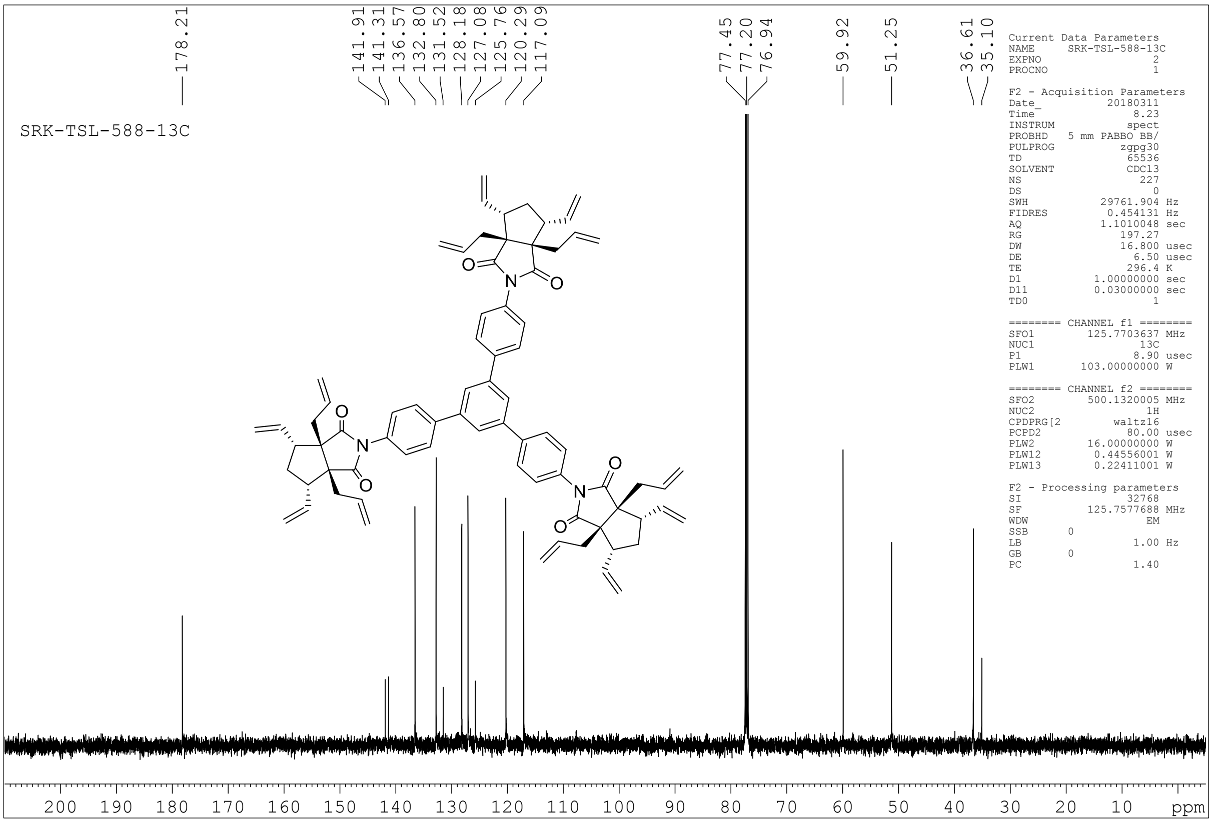

HRMS of Compound 14

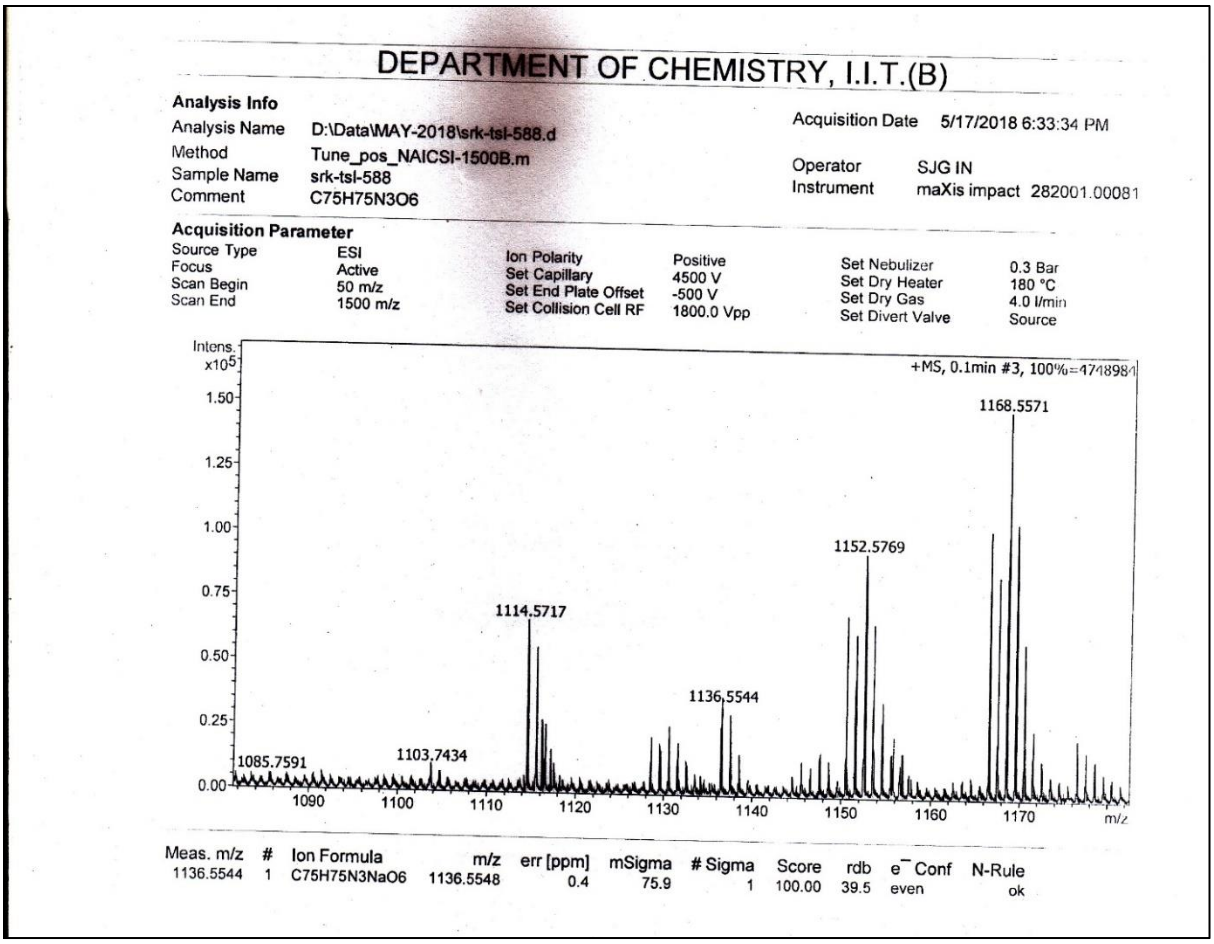

DEPARTMENT OF CHEMISTRY, I.I.T.(B)

Analysis Info

Analysis Name	D:\Data\MAY-2018\srk-tsl-588.d	Acquisition Date	5/17/2018 6:33:34 PM
Method	Tune_pos_NAICSI-1500B.m		
Sample Name	srk-tsl-588	Operator	SJG IN
Comment	C75H75N3O6	Instrument	maXis impact 282001.00081

Acquisition Parameter

Source Type	ESI	Ion Polarity	Positive	Set Nebulizer	0.3 Bar
Focus	Active	Set Capillary	4500 V	Set Dry Heater	180 °C
Scan Begin	50 m/z	Set End Plate Offset	-500 V	Set Dry Gas	4.0 l/min
Scan End	1500 m/z	Set Collision Cell RF	1800.0 Vpp	Set Divert Valve	Source

Meas. m/z	#	Ion Formula	m/z	err [ppm]	mSigma	# Sigma	Score	rdb	e⁻ Conf	N-Rule
1136.5544	1	C75H75N3NaO6	1136.5548	0.4	75.9	1	100.00	39.5	even	ok

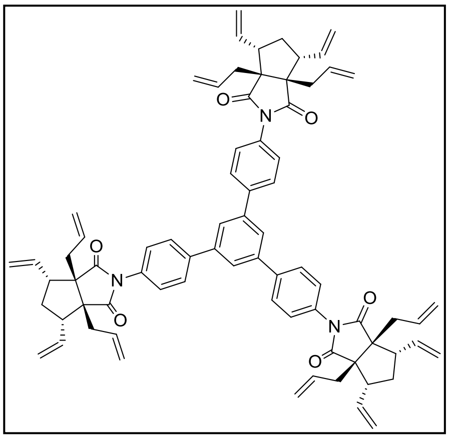

^{13}C NMR of compound 15 in $CDCl_3$

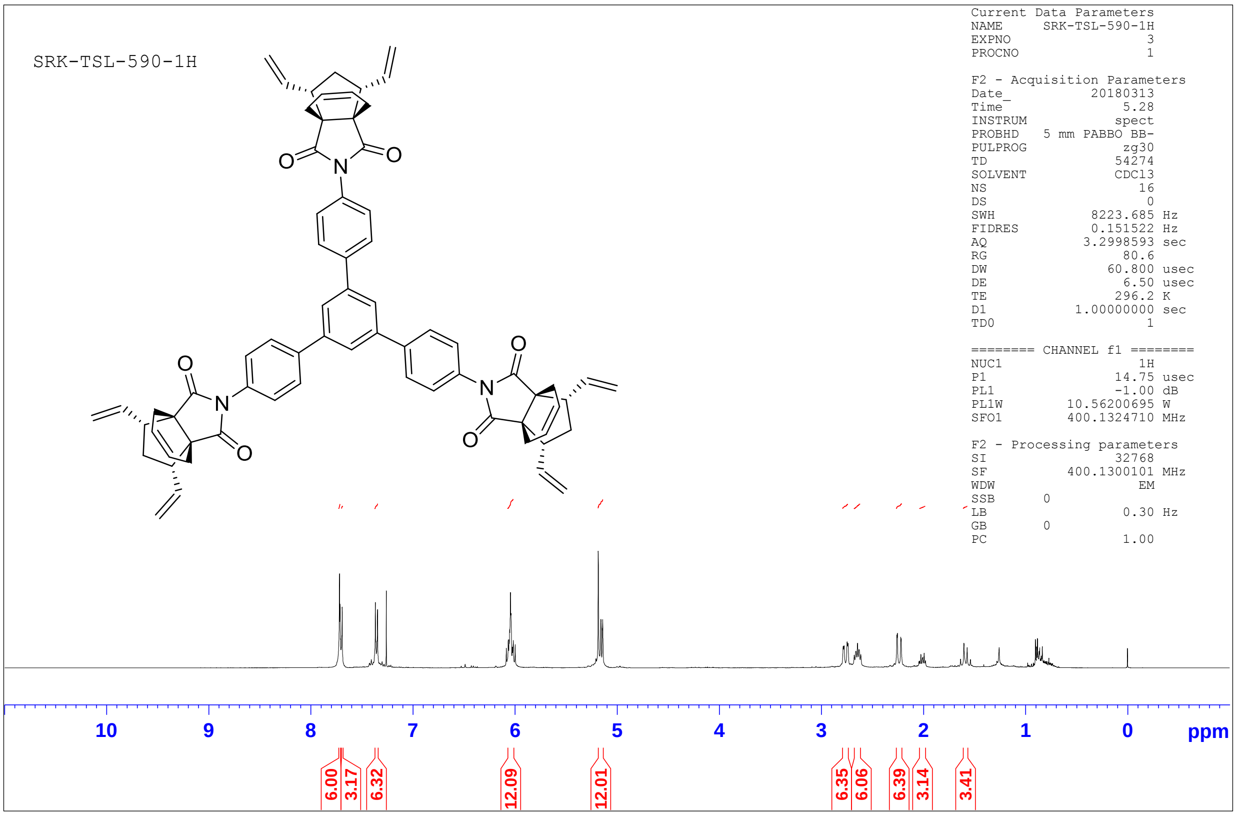

^{13}C NMR of compound 15 in $CDCl_3$

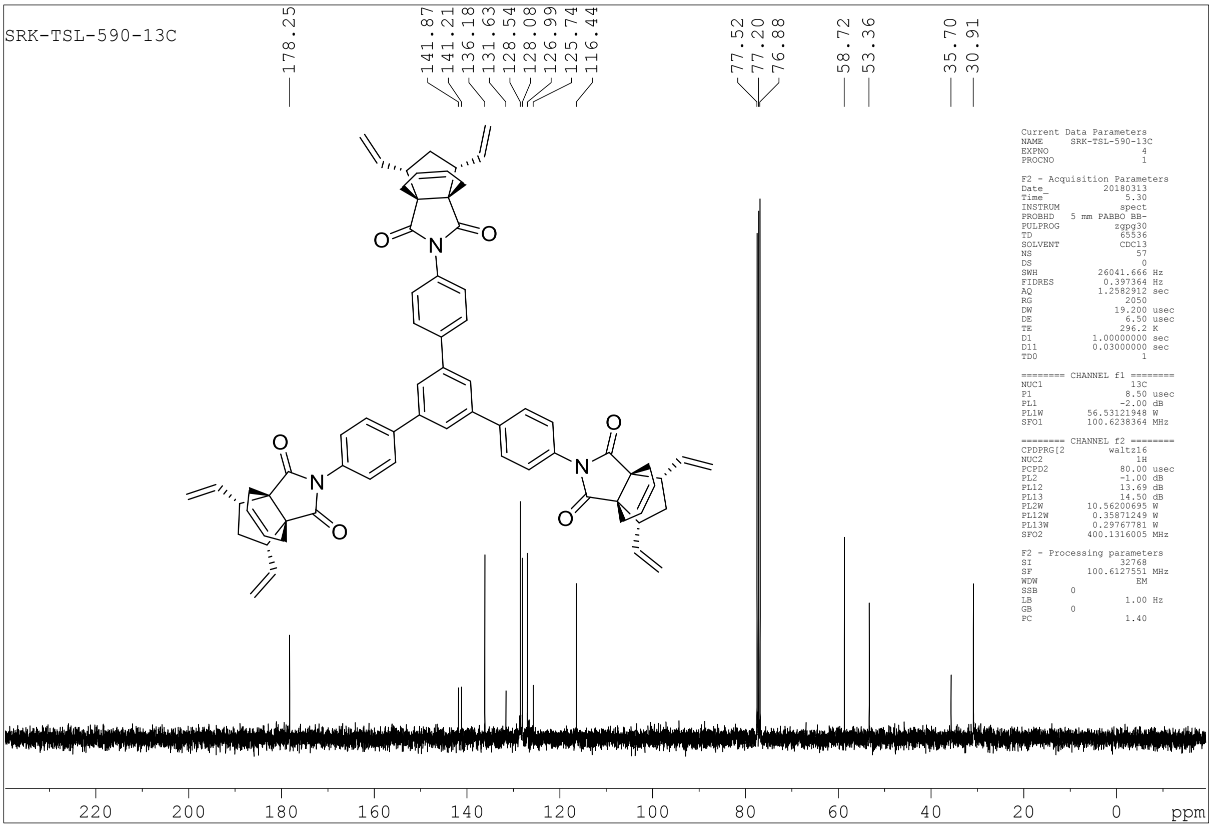

HRMS of Compound 15

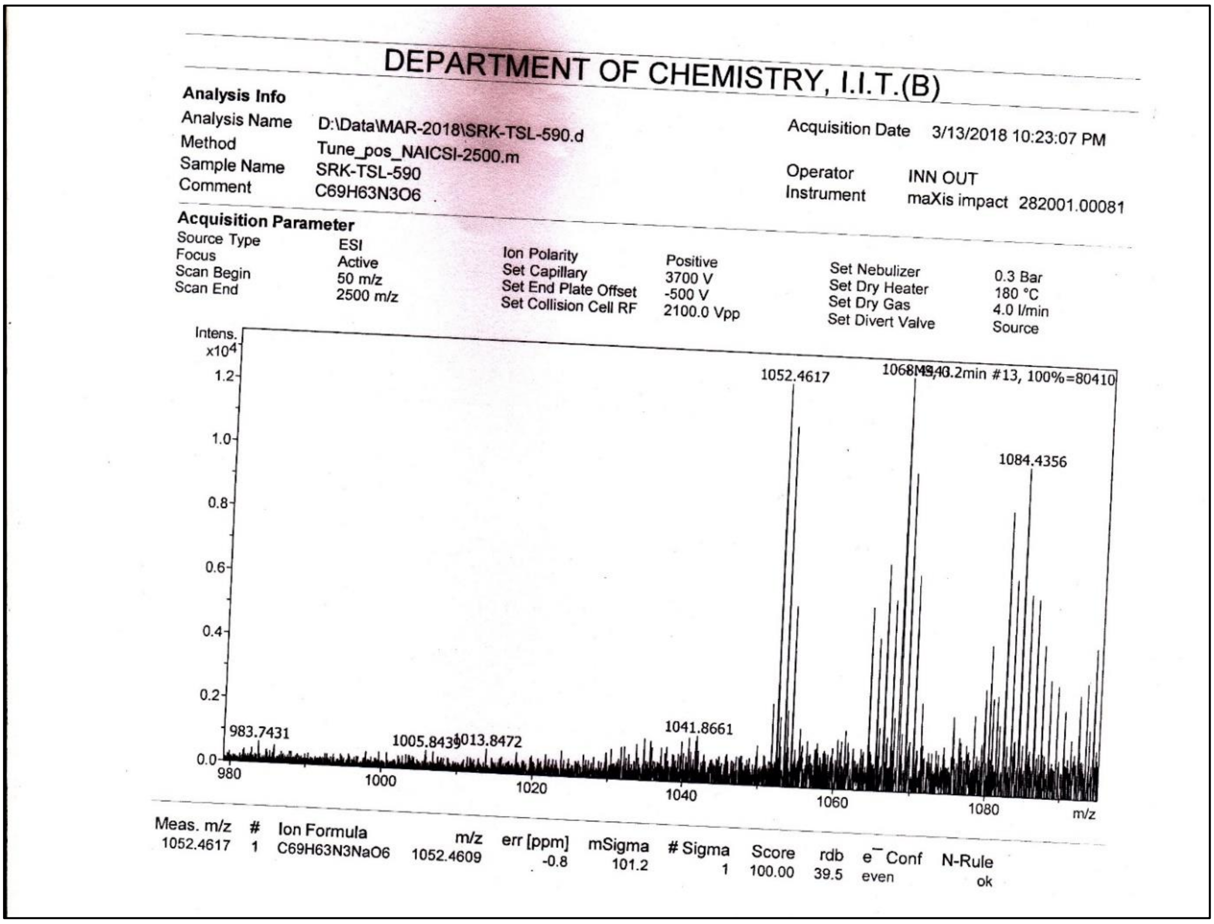

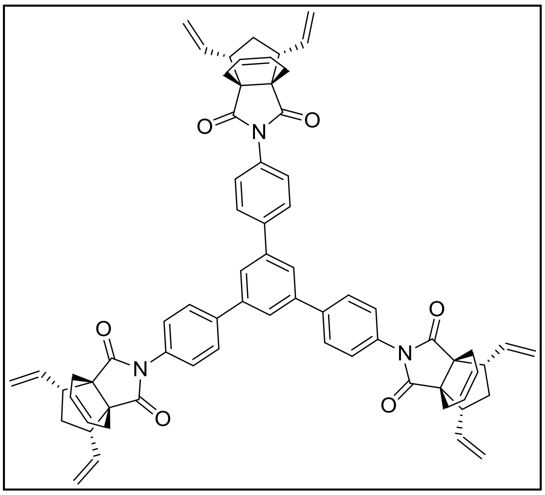

^{13}C NMR of compound 21 in $CDCl_3$

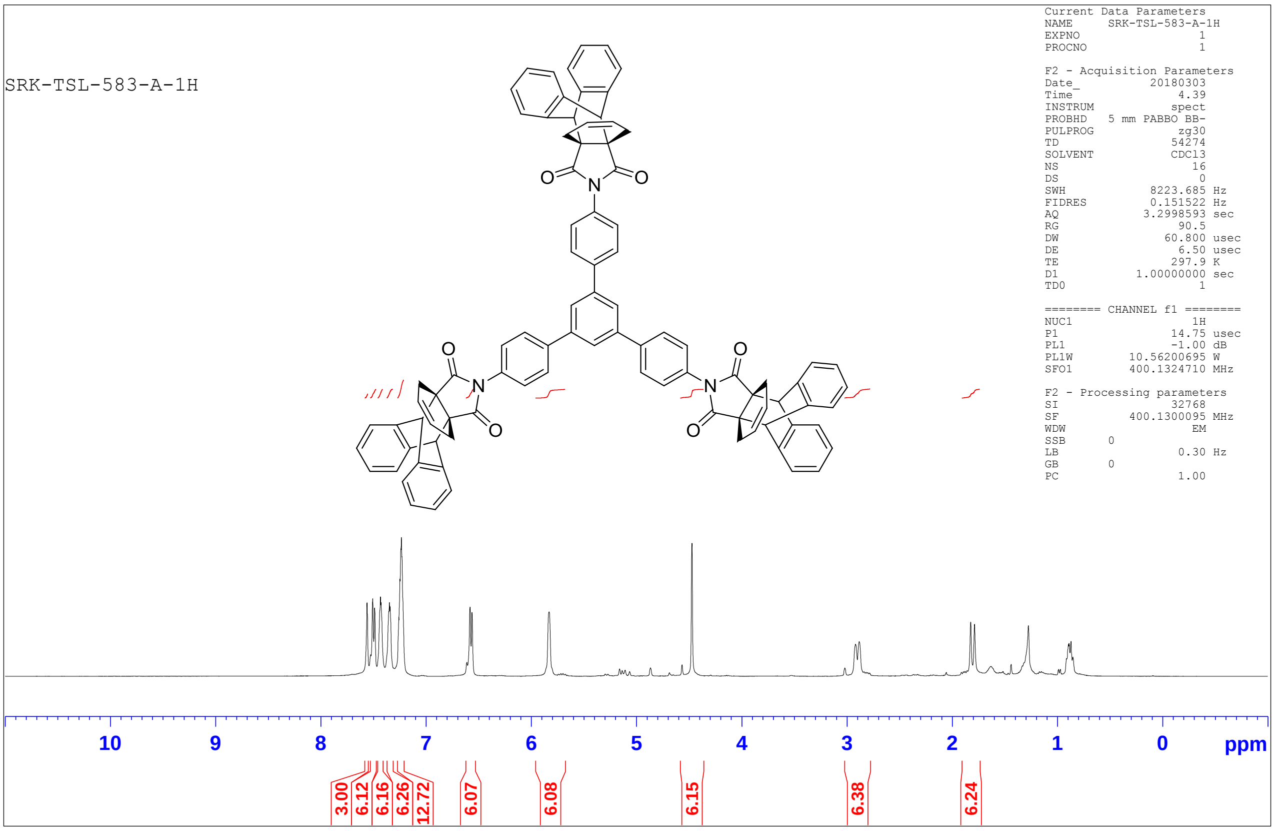

^{13}C NMR of compound 21 in $CDCl_3$

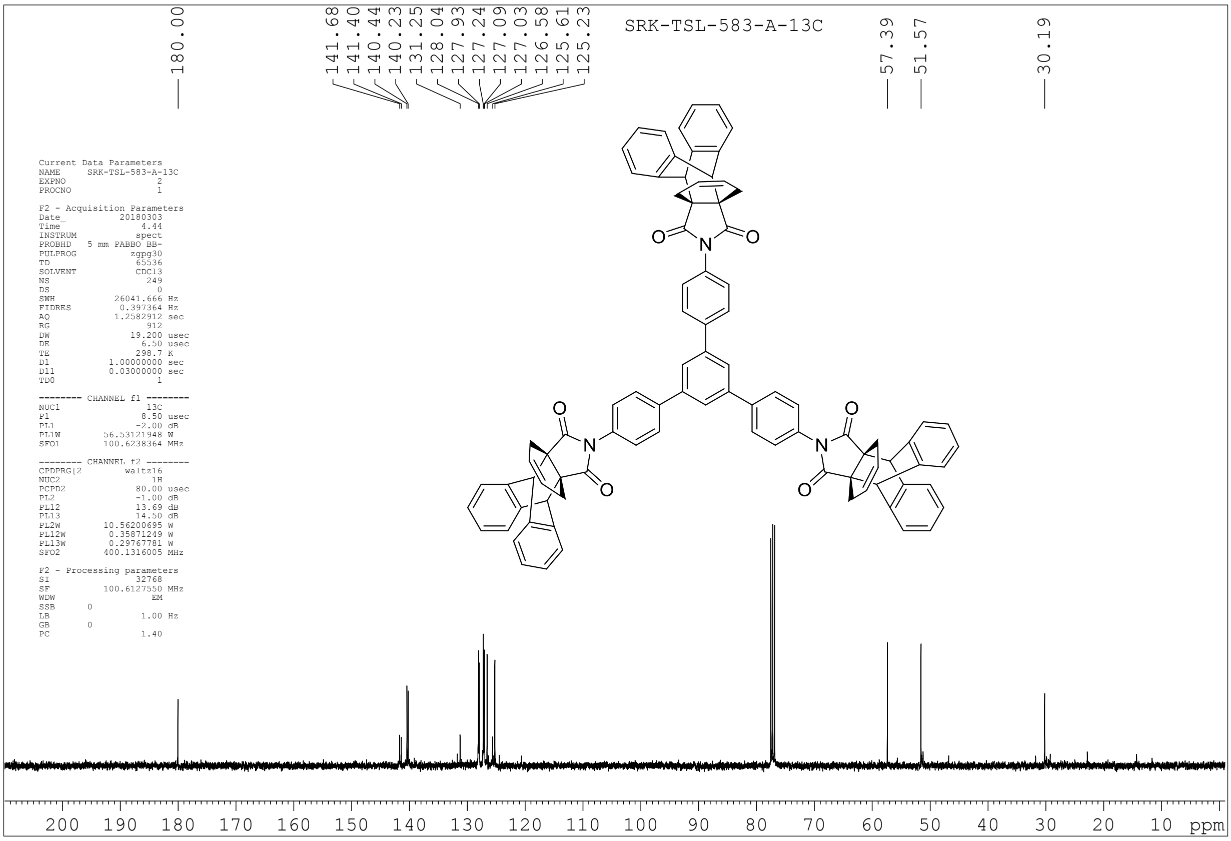

HRMS of Compound 21

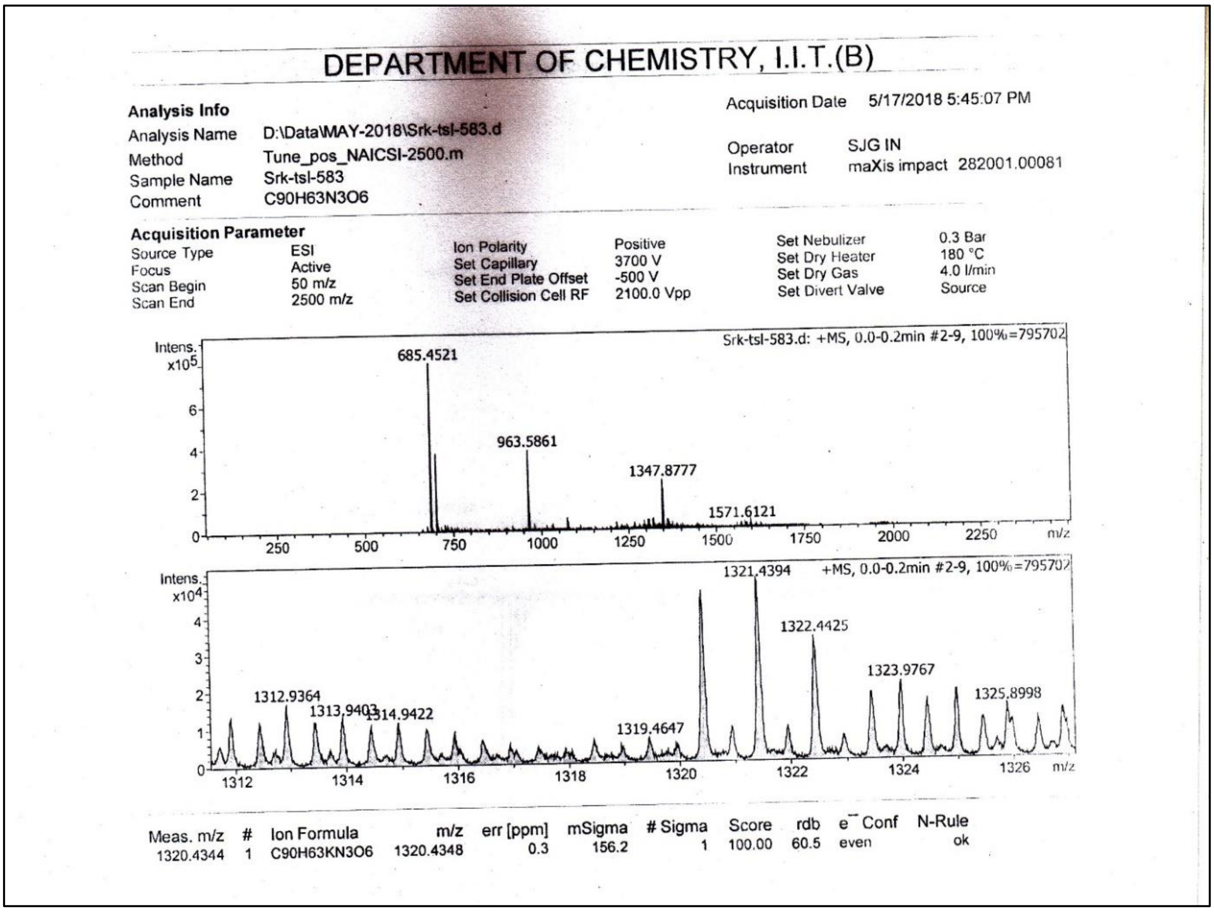
DEPARTMENT OF CHEMISTRY, I.I.T.(B)

Analysis Info

Analysis Name D:\Data\MAY-2018\Srk-tsl-583.d

Method Tune_pos_NAICSI-2500.m

Sample Name Srk-tsl-583

Comment C90H63N3O6

Acquisition Date 5/17/2018 5:45:07 PM

Operator SJG IN

Instrument maXis impact 282001.00081

Acquisition Parameter

Source Type	ESI	Ion Polarity	Positive	Set Nebulizer	0.3 Bar
Focus	Active	Set Capillary	3700 V	Set Dry Heater	180 °C
Scan Begin	50 m/z	Set End Plate Offset	-500 V	Set Dry Gas	4.0 l/min
Scan End	2500 m/z	Set Collision Cell RF	2100.0 Vpp	Set Divert Valve	Source

Meas. m/z	#	Ion Formula	m/z	err [ppm]	mSigma	# Sigma	Score	rdb	e⁻ Conf	N-Rule
1320.4344	1	C90H63KN3O6	1320.4348	0.3	156.2	1	100.00	60.5	even	ok

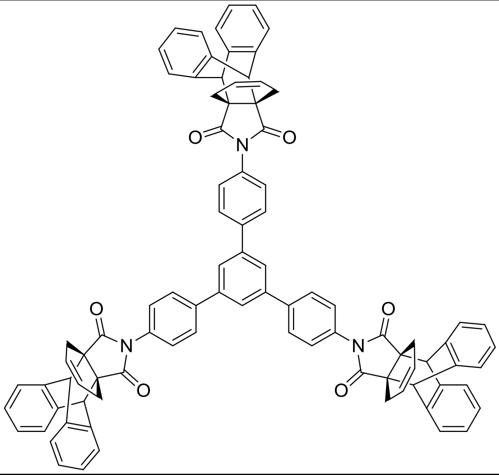

Supplement: File 1 — Copies of 1H, 13C NMR and HRMS spectra of new compounds. [file Beilstein_J_Org_Chem-14-2537-s001.pdf]
